# Light-Harvesting Crystals Formed from BODIPY-Proline Biohybrid Conjugates: Antenna Effects and Excitonic Coupling

**DOI:** 10.1021/acs.jpca.2c00035

**Published:** 2022-03-01

**Authors:** Sara M. Waly, Joshua K. G. Karlsson, Paul G. Waddell, Andrew C. Benniston, Anthony Harriman

**Affiliations:** ^†^Molecular Photonics Laboratory and ^‡^Crystallography Laboratory, School of Natural & Environmental Sciences, Newcastle University, Bedson Building, Newcastle upon Tyne NE1 7RU, U.K.

## Abstract

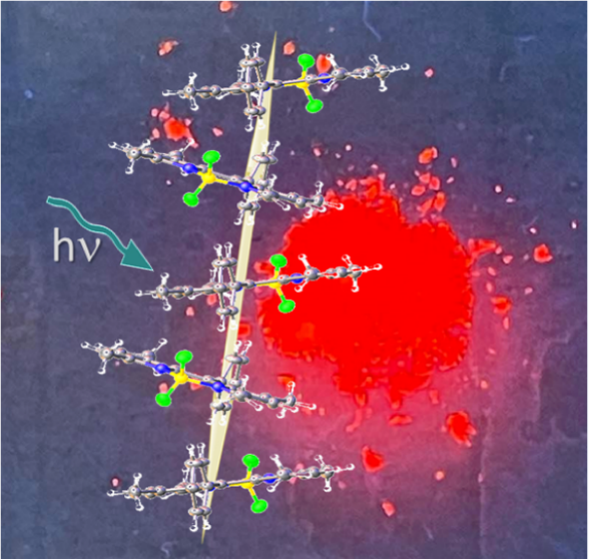

A boron dipyrromethene (BODIPY) derivative bearing a *cis*-proline residue at the *meso*-position crystallizes in the form of platelets with strong (i.e., Φ_F_ = 0.34) red fluorescence, but the absorption and emission spectra differ markedly from those for dilute solutions. A key building block for the crystal is a *pseudo*-dimer where hydrogen bonding aligns the proline groups and separates the terminal chromophores by ca. 25 Å. Comparison with a covalently linked bichromophore suggests that one-dimensional (1D) excitonic coupling between the terminals is too small to perturb the optical properties. However, accretion of the *pseudo*-dimer forms narrow channels possessing a high density of chromophores. The resultant absorption spectrum exhibits strong excitonic splitting, which can be explained quantitatively using the extended dipole approach and allowing for coupling between ca. 30 BODIPY units. Fluorescence, which decays with a lifetime of 2.2 ns, is assigned to a delocalized and (slightly) super-radiant BODIPY dimer situated at the interface and populated via electronic energy transfer from the interior.

## Introduction

Photosynthesis is the generic term used to describe the numerous disparate ways in which natural organisms use sunlight to generate a useful chemical fuel that can be stored until required.^1^ In all cases, light-harvesting complexes (LHCs) are utilized^2^ to gather more of the incident sunlight than could be collected by the isolated catalytic site responsible for redox chemistry. The composition and structure of LHCs diverge markedly among the various organisms, but they share a common function: namely, the collection of sunlight over discrete wavelength ranges and the subsequent transfer of the excitation energy to a specific reaction site.^3−5^ Interest in natural LHCs has stimulated the growth of artificial analogues in which various chromophores have been assembled in a logical sequence likely to ensure electronic energy transfer (EET) along a thermodynamic gradient.^6−9^ Initial systems^10,11^ were based on covalently linked structures formed from multiple disparate chromophores, labeled dendrimers^12^ being a prime example. Such materials are readily studied by time-resolved spectroscopy so that the dynamics of intramolecular EET can be measured and related to the molecular architecture. Many imaginative arrays have been introduced along these lines, but while being both elegant and synthetically challenging,^13,14^ they lack the photon collection capability of natural systems. This is because only a handful of chromophores can be assembled into a cooperative network using this approach,^15^ and this does not provide for sufficient light collection. Noncovalent interactions can be invoked^16^ to engineer larger accretions of chromophores but often give rise to strong electronic interactions that perturb the molecular properties; J-aggregates formed from cyanine dyes^17^ might be considered as a typical example. Alternative structures, such as polymers^18^ or quantum dots,^19,20^ might allow close packing of multiple chromophores without affecting the photophysical properties but present a formidable challenge in terms of arranging for the logical cascade of EET steps considered necessary for panchromatic light collection.

The search for new types of artificial LHCs has led to the identification of certain fluorescent crystals.^21−24^ This is perhaps a surprising choice of material since close contact between neighboring molecules in the crystal lattice is expected to ensure efficient excited-state deactivation by way of proximity quenching. Additional problems that might be anticipated include self-absorption,^25^ caused by high local concentrations, and quenching via defect states or dislocations. Nonetheless, there is a steadily growing number of cases where strong emission can be observed from single crystals comprising simple organic chromophores.^26,27^ Such systems solve the synthetic problems related to covalently linked arrays and have the added attraction that they can be dismantled, purified to remove damaged components, and reassembled. Furthermore, it should be possible to identify crystals possessing well-defined tracts suitable for unusually long-range EET processes. This latter situation is difficult, if not impossible, to arrange with other types of artificial LHCs. The main problem related to the development of light-harvesting crystals concerns the lack of clear guidelines on how to hinder proximity quenching while encouraging directional EET over hundreds of chromophores.

Most attempts to identify strongly luminescent crystals have focussed on the chromophore, a common strategy being to include bulky side groups to help minimize close contacts. Here, we take a different approach whereby the chromophore, this being a boron dipyrromethene (BODIPY) dye,^28−30^ is equipped with a side chain known to self-associate into ordered structures. Our first such example has a short proline residue attached at the *meso*-phenyl ring of the BODIPY chromophore. It was anticipated that secondary interactions between proline groups might aid the formation of ordered domains that help isolate the BODIPY chromophores. The crystal structure derived from X-ray crystallography indicates that this hypothesis is essentially correct, with the BODIPY units aligning as filaments, or wires, which act as light guides.^31^ Excitonic coupling^32^ between nearby BODIPY residues serves to broaden the absorption spectrum, thereby increasing the light collection capacity without seeming to promote radiationless decay.

## Experimental Section

### Synthesis and Compound Characterization

**Scheme 1:**
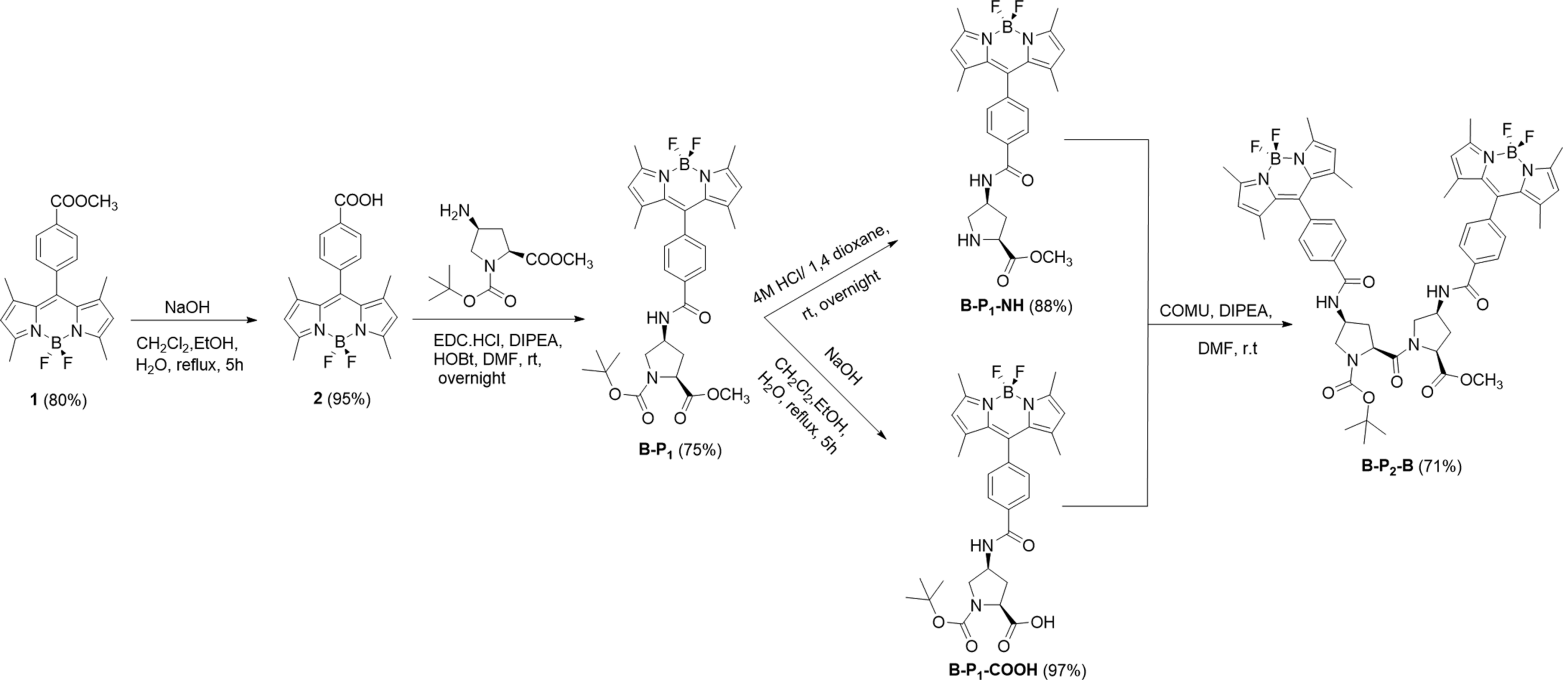
Synthetic Procedures for the Preparation of the Target Proline-Based BODIPY Chromophores B-P_1_, B-P_1_-NH, B-P_1_-COOH, and B-P_2_-B

Molecular formulas and synthetic routes to the target compounds B-P_1_ and B-P_2_-B are outlined in Scheme 1. Preparation of **1** was reported previously,^33^ but the yield could be increased to 80% using *N*,*N*-diisopropylethylamine as the base rather than trimethylamine since this prevented decomposition and side product formation. The attempted hydrolysis of ester **1** with KOH, LiOH, or NaOH in a polar solvent mixture (CH_2_Cl_2_/C_2_H_5_OH/H_2_O) afforded **2** in yields of 60, 70, and 95%, respectively. The target B-P_1_ was prepared in 75% yield by coupling the carboxylic acid derivative **2** with the *cis*-proline derivative **3** using EDC·HCl^34^ and HOBt^35^ in dimethylformamide (DMF) (Scheme 1). The subsequent deprotection of B-P_1_ was attempted using TFA/CH_2_Cl_2_ (1/1), but this was unsuccessful, resulting in a complex mixture of products. However, a significant improvement in product isolation was obtained using 4M HCl in 1,4-dioxane, which afforded B-P_1_-NH in 88% yield (Scheme 1). The hydrolysis of B-P_1_ was achieved using 50 equivalents of NaOH in a mixture of CH_2_Cl_2_/CH_3_OH/H_2_O (3/3/0.5) (Scheme 1). The synthesis of the symmetrical dimer B-P_2_-B was achieved in 71% yield by coupling B-P_1_-NH and B-P_1_-COOH using COMU^36^ as the peptide coupling reagent in the presence of *N*,*N*-diisopropylethylamine as a base in DMF at room temperature (Scheme 1).

All compounds were fully characterized by conventional analytical techniques including high-field NMR spectroscopy focussing on ^1^H, ^13^C, ^11^B, and ^19^F nuclei. Other verification methods included high-resolution mass spectrometry (HRMS) and X-ray diffraction analyses (Figures S1–S16). The successful synthesis of B-P_1_ was confirmed from its ^1^H NMR spectrum (see the Supporting Information) by the appearance of the signal for *Boc* (i.e., *tert*-butyloxycarbonyl) methyl protons at 1.37 ppm, as well as broad signals in the range between 2.03 and 4.83 ppm, which are readily assigned to the proline aliphatic CH_2_/CH protons. Confirmation of the molecular composition of B-P_1_ was obtained by positive electrospray ionization (ESI) mass spectrometry (CH_2_Cl_2_/CH_3_OH + NH_4_OAc), which displayed a peak at *m*/*z* = 617.2723 corresponding to the [M + Na]^+^ ion and a peak at *m*/*z* = 595.2903 corresponding to the [M + H]^+^ ion.

### X-ray Crystallography

Suitable crystals were grown through slow evaporation of a solution of B-P_1_ in hexane/CH_2_Cl_2_ (3/1). Single-crystal diffraction data were collected at 150 K on an Xcalibur, Atlas, Gemini ultradiffractometer equipped with an Oxford Cryosystems CryostreamPlus open-flow N_2_ cooling device using copper X-radiation (λ_Cu Kα_ = 1.54184 Å). Intensities were corrected for absorption empirically using spherical harmonics. Cell refinement, data collection, and data reduction were undertaken via software CrysAlisPro.^37^ Structures were solved using XT^38^ and subsequently refined by XL^39^ using the Olex2 interface.^40^ All nonhydrogen atoms were refined anisotropically, and hydrogen atoms were positioned with idealized geometry, except for those bound to heteroatoms, the positions of which were located using peaks in the Fourier difference map. The displacement parameters for hydrogen atoms were constrained using a riding model with *U*_(H)_ set to be an appropriate multiple of the *U*_eq_ value of the parent atom. Geometry information used for the various calculations was extracted using the Olex2 interface. Center-to-center distances were determined using a dummy atom inserted midway between the boron and *meso*-carbon atoms.

### Spectroscopic Studies

Absorption spectra were recorded with a Hitachi U3310 spectrophotometer, while emission spectra were recorded with a Hitachi F4500 fluorescence spectrophotometer. Liquid phase spectra were recorded for optically dilute solutions using a small range of concentrations to ensure linearity of the instrument response. After performing any baseline corrections, data were transferred to a microcomputer for subsequent analysis using purpose-written software. Absorption spectra for solid-state samples used the same instrument but with crystals adhered to quartz microscope slides with transparent tape. Slides were cleaned by soaking in concentrated hydrochloric acid for at least 4 h, before being washed with copious amounts of deionized water followed by methanol and then allowed to dry. Several such slides were used for each measurement, with the slide being repositioned in the light beam. Fluorescence studies were made with a home-built three-dimensional (3D) positional stage for manipulating the crystalline sample within the excitation beam to minimize scattered and/or reflected light. Cutoff filters were used to prevent excitation light reaching the detector. A variety of excitation wavelengths was employed, and all emission spectra were supported by excitation spectra. Fluorescence quantum yields were measured^41^ for dilute solutions, for which the mean absorbance at the excitation wavelength was adjusted to ca. 0.05, by comparison to recognized standards recorded^42^ under identical conditions. The same solution was used to record the emission lifetime using a PTI Time-Master single-photon counting setup. Excitation was made at 505 nm with a pulsed light-emitting diode (LED) (FWHM = 0.30 ns), and emission was detected at 550 ± 5 nm with a fast response photomultiplier tube (PMT). Analysis was made by standard statistical methods.^43^

Fluorescence quantum yield measurements for single crystals were made with the sample held in an integrating sphere (StellaNet IS6). Excitation was made with a narrow bandwidth LED transmitted through a diffusive quartz plate to remove polarization and to attenuate the light intensity. Output from the integrating sphere was collected with an Olympus microscope U-UCLHG/XEA collector lens and directed to the spectrophotometer using fiber optics. Glass cutoff filters were used to remove any scattered light. Analysis followed the procedure recommended by Beeby et al.^44^ Fluorescence lifetime measurements were made by time-correlated, single-photon counting with excitation at either 440, 505, or 525 nm. The sample was supported on a quartz plate and positioned in the incident light beam. Glass cutoff filters were used to minimize reflected excitation light, and emission was passed through a series of narrow band-pass filters to isolate required spectral regions. All measurements were repeated several times.

Geometry optimizations of B-P_1_ and B-P_2_-B were performed with the GAMESS program^45^ with the 6-311G(2d,p) basis set^46^ and the PBE0 functional.^47^ Initial studies were made for the ground-state molecule in the gas phase, and the accompanying frequency calculations were made using the same functional. The contribution of solvent effects was taken into account using the CPCM-BMK/6–311(2d,p) basis set.^48,49^ The solvent for these studies was chloroform. Normal mode analyses for each structure yielded no imaginary frequencies for the 3*N* – 6 vibrational degrees of freedom, where *N* is the number of atoms in the system. This finding is taken to indicate that the structure of each molecule corresponds to at least a local minimum on the potential energy surface.

## Results and Discussion

Photosynthetic organisms have evolved in such a way that light harvesting is spatially isolated from fuel production,^1^ in some cases by as much as 1,000 Å. This simple strategy allows separate optimization of the various components, with photon collection being assigned to pigment–protein complexes localized as an antenna unit.^2−6^ Despite the large spatial separation, the reaction center is supplied with a constant stream of photons by way of rapid EET between pigments and ultimately to the reaction center. An individual photon might sample hundreds, if not thousands, of chromophores before being irreversibly trapped at the reaction center. In contrast, artificial LHCs comprising covalently linked chromophores are restricted to around 20 or so absorbers^10,11^ and therefore lack the required photon collection capacity.^15^ This investigation considers the case for replacing the covalently linked entity with crystal possessing channels, which themselves are richly packed with chromophores arranged in the form of filaments.

### Synthesis

Inspired by natural LHCs, a large number of artificial analogues have been synthesized^10−15^ in which sequential EET serves to shuttle the exciton around the molecular edifice. An important, but often overlooked, aspect of these multicomponent entities is the choice of spacer group used to isolate the chromophores. These spacers are usually multiples of small rigid modules, such as phenyl or tolane, incremented to give the required separation distance and/or orientation between the chromophores. Longer spacers are prone to twisting and bending forces that shorten the molecular length relative to the fully extended structure,^50,51^ even when individual connectors are rigid. Alternatively, the use of flexible spacers can be complicated by a wide distribution of conformations.^52^ Herein, we use a spacer that might be expected to control the molecular structure by the close association between spacers, aided by intermolecular hydrogen bonding. The intention is to increase light collection by bringing together many chromophores but in a nonrandom manner that dispels proximity quenching.

An important feature of the target compound, hereafter abbreviated as B-P_1_ (Scheme 1), concerns the amide connection, which is partially conjugated to the phenyl ring. The proline unit, which is nonplanar, exists in the *cis*-geometry with respect to the substituents, at least for the crystalline sample. The ^1^H NMR spectrum of B-P_1_-NH is useful in confirming the absence of the *Boc* leaving group; unequivocal confirmation of the molecular composition was obtained from the appearance of a prominent peak at *m*/*z* = 517.2198 in the positive ESI mass spectrum. The disappearance of methyl ester protons at 3.72 ppm for B-P_1_-COOH confirms hydrolysis of the ester group. The synthesis of the symmetrical dimer B-P_2_-B was confirmed by the presence of peaks at 1.45 ppm ascribable to the *Boc* group and the observation of the ester protons at 3.74 ppm.

For this compound, where unequivocal confirmation of the molecular composition arises from the major peak at *m*/*z* = 1037.4881 in the positive ESI mass spectrum, there is an important change in the integration for the proline protons. These materials are readily soluble in common organic solvents and stable on prolonged storage at room temperature. Full details for the characterization of new compounds are provided in the Supporting Information (Figures S1–S16).

### Optical Properties in Fluid Solution

**Figure 1:**
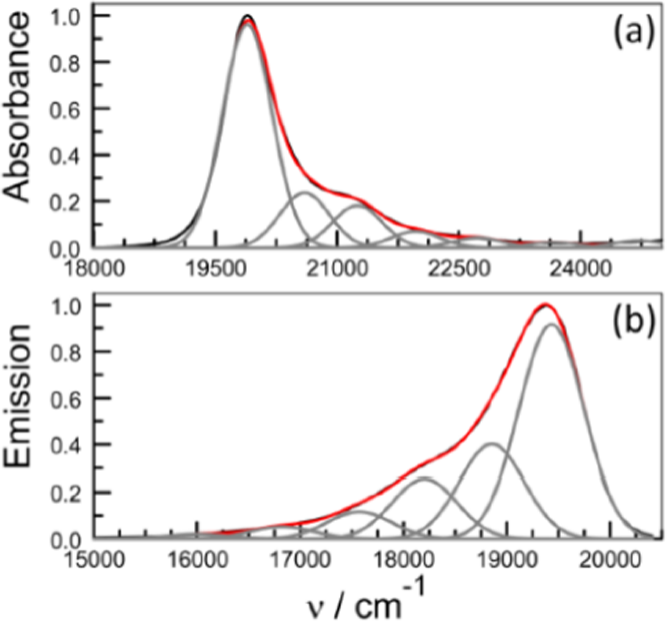
(a) Reduced absorption and (b) reduced fluorescence spectra compiled for B-P_1_ in CH_2_Cl_2_ solution. The experimental curve is shown in black, while the simulated spectrum is overlaid in red. Individual Gaussian components are shown as gray curves.

Absorption and fluorescence spectra recorded for B-P_1_ in CH_2_Cl_2_ solution display the features considered characteristic of conventional BODIPY derivatives (Figure S20).^28−30^ The absorption spectrum is relatively narrow, the reduced spectrum^43^ having a full width at half-maximum (FWHM) of 680 cm^–1^, with a well-defined peak centered at 503 nm (Figure 1). The first-allowed absorption transition shows a vibronic progression with a spacing (*h*ω_M_) of 640 cm^–1^. Absorption to higher excited states is evident at around 400 nm. The emission spectrum has a maximum at 516 nm (Figure S20), while the FWHM of the reduced spectrum is 770 cm^–1^ (Figure 1). Reasonable mirror symmetry is observed with the absorption profile, and the emission profile has a vibronic spacing of 660 cm^–1^. The Huang–Rhys factor^53^ (*S*_M_) for the reduced fluorescence spectrum is 0.36. From this information, the reorganization energy (*L*) for excitation and relaxation of the monomer in solution is calculated to be 250 cm^–1^ from the Stokes shift^54^ or 260 cm^–1^ from spectral deconstruction.^55^ Integration of the reduced emission spectrum allows determination^56^ of the transition dipole moment (μ_TD_) as being 5.4 ± 0.3 D, while the corresponding value obtained^57^ from the absorption spectrum is 4.9 ± 0.4 D (see the Supporting Information).

The fluorescence quantum yield (Φ_F_) in CH_2_Cl_2_ was found to be 0.62 ± 0.03, while the emission lifetime (τ_S_), derived by time-correlated, single-photon counting, is 4.8 ± 0.2 ns (Figure S24). These values are comparable to those reported for other conventional BODIPY derivatives,^28−30^ thereby indicating that the proline group does not impose a new nonradiative deactivation step.^58^ It is notable that there is no indication of electronic coupling between the chromophore and proline residue in the solution. The other important point to note is that the radiative rate constant (*k*_RAD_ = 1.3 × 10^8^ s^–1^) remains in line with related BODIPY derivatives.^28−30^

**Figure 2:**
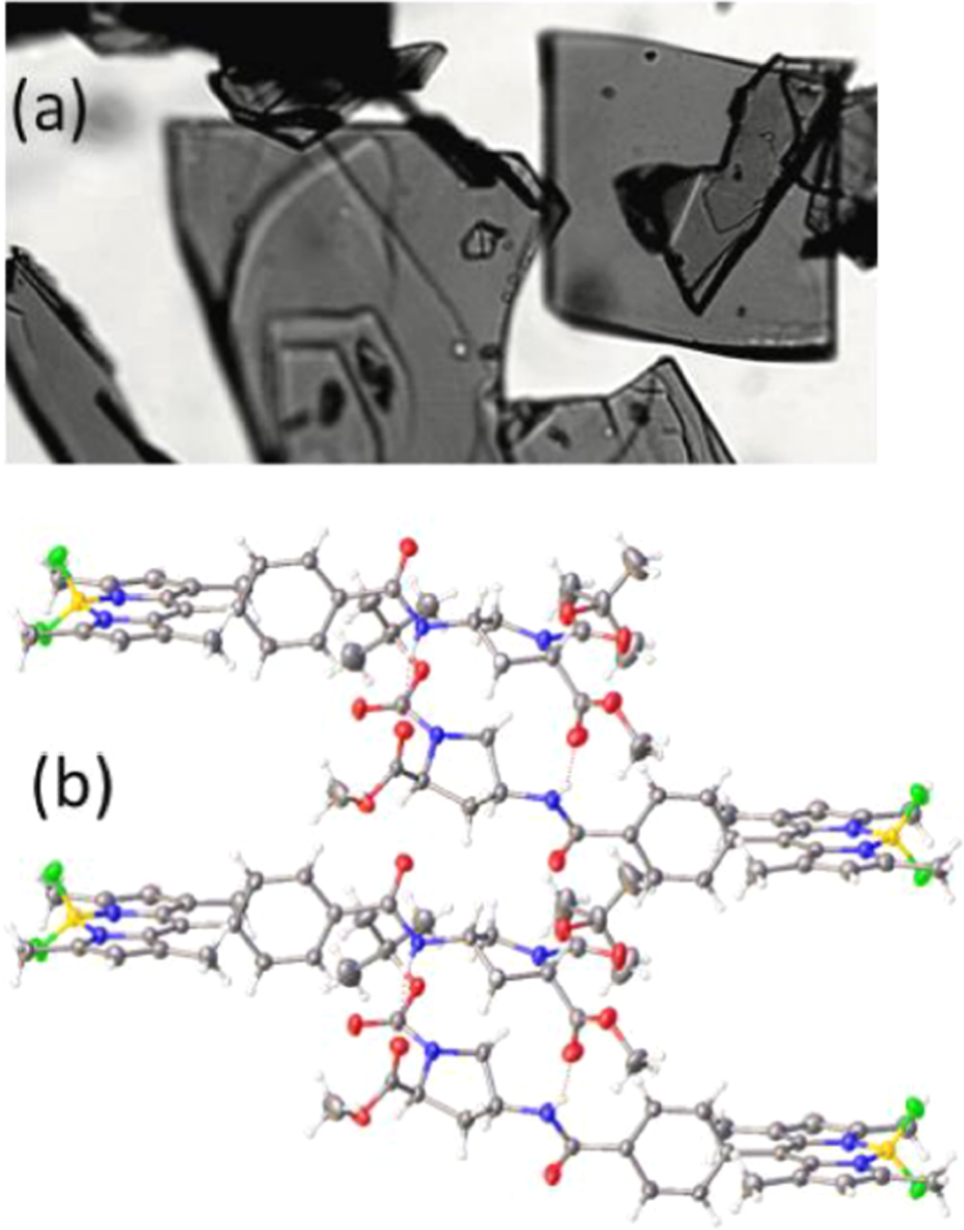
(a) Photograph of crystals of B-P_1_ taken with an optical microscope. (b) Image taken from the X-ray crystal structure determined for B-P_1_ highlighting the so-called *pseudo*-dimer assembled by way of two hydrogen bonds (shown as red dashed lines) between the stacked proline residues. Two such species assemble so as to align the terminal BODIPY units in a cofacial arrangement that is the building block for formation of columns (or filaments) rich in chromophore.

We have identified a hydrogen-bonded dimer as being the basic building block for the crystal (Figure 2). To examine the likelihood of excitonic coupling between the two BODIPY terminals, we have synthesized the covalently linked dimer, B-P_2_-B, as a control (Scheme 1). The absorption and emission spectra recorded for this compound in CH_2_Cl_2_ (Figure S21) remain similar to those observed for B-P_1_, while it is of particular significance to note there is no broadening or splitting of the lowest-energy absorption transition. Both absorption (λ_ABS_ = 504 nm) and fluorescence (λ_FLU_ = 517 nm) peak maxima remain comparable to those of the control while the FWHMs derived from reduced spectra are 710 and 715 cm^–1^, respectively (Figure S25). The Huang–Rhys factor (*S*_M_) determined for the symmetrical dimer is 0.37.

The computed (DFT/CPCM-BMK/6–311(2d,p)/PBE0) molecular structure for B-P_1_ is in quite good agreement with that determined by X-ray crystallography (Figure S18). The same level of calculation carried out for the dimer, B-P_2_-B, indicates that the two proline groups are twisted but keep the BODIPY units well separated (Figure S19). Indeed, the center-to-center separation distance is calculated to be ca. 20.9 Å, while the mutual angle between the respective transition dipole moment vectors is ca. 20.3° (Figure S22). Both *meso*-phenyl rings adopt a dihedral angle of ca. 57° with respect to the plane of the adjacent BODIPY chromophore. Of the two amide groups, one exists in the *trans* conformation while the other adopts the *cis* configuration. This structural motif is set in part by the bulky *Boc* protecting group and the methyl ester substituent, both of which are directed away from the *bis*-proline spacer. There are two eight-member hydrogen bonds, with O...H distances of ca. 2.7 Å, involving the N–H groups and complementary C=O groups. These hydrogen bonds help establish the molecular structure around the spacer. The two proline units are positioned in an almost orthogonal (i.e., 78° twist) arrangement that shortens the molecular axis compared to a fully extended geometry.

Making use of Kasha theory^59^ for an oblique pairing, the degree of excitonic coupling (*J*_D_) between the two BODIPY terminals is calculated to be −22 ± 3 cm^–1^ (Figure S22). Because the two BODIPY planes are almost parallel and relatively far apart, the orientation factor^60^ needed for this calculation is subject to error. A more reliable determination of the degree of excitonic interaction between the BODIPY units can be made using the extended dipole protocol introduced by Kuhn et al.,^61,62^ which makes use of the distances between individual atoms rather than relying on mutual angles (Figure S23). This approach leads to a coupling element of ±12.5 ± 1.5 cm^–1^, which is in reasonable agreement with that determined from Kasha theory but perhaps is more precise. The derived coupling element is negligible compared to the FWHM for the absorption transition such that it will not perturb the experimental absorption spectrum recorded for B-P_2_-B in CH_2_Cl_2_ solution. Repeating the calculation for the *pseudo*-dimer depicted in the X-ray structure indicates that excitonic coupling between the terminals will be on the order of −15 ± 2 cm^–1^ (Kasha theory) or ± 12 ± 1 cm^–1^ (Kuhn extended dipole method). Again, this small interaction energy is unlikely to affect the absorption spectrum.

For the symmetrical dimer, B-P_2_-B, the spectral overlap integral (ψ) between reduced absorption and reduced fluorescence profiles is calculated^63^ to be 1.4 ± 0.2 × 10^–4^ cm. This value is hardly affected by changes in the solvent. The square of the corresponding orientation factor (κ^2^) has a value of 0.73. Applying the transition dipole moments calculated earlier, the rate constant (*k*_DD_) for resonance energy transfer between the terminal BODIPY units is calculated to be 7 × 10^8^ s^–1^ for a center-to-center separation of 20.9 Å (see the Supporting Information). Comparing this value with the inherent excited-state lifetime (τ_S_) of 4.8 ns leads to the conclusion that one-dimensional (1D) exciton migration between the terminals is slightly faster than emission. This would allow the exciton to alternate several times between the terminals before deactivation.

### X-ray Crystal Structure Derived for B-P_1_

Flat, platelike crystals were grown through slow evaporation of a solution of B-P_1_ in hexane/CH_2_Cl_2_ (3/1) (Figure 2a). A subsequent crystal structure determination, carried out at 150 K, confirmed both the authenticity of the sample and that attachment to the BODIPY residue does not affect the *cis* arrangement around each proline group.^64^ Under illumination with UV light, the crystals emit red fluorescence (see ToC graphic). The crystal system is orthorhombic with the P2_1_2_1_2_1_ space group. The structure derived for a single B-P_1_ molecule, together with the atom numbering system, is illustrated by way of Figure S17, and the pertinent structural data are given in Tables S1–S8. The calculated density is 1.28 g/cm^3^. The amide group persists in the *trans* configuration, while the two ester groups are directed away from the BODIPY chromophore. The amide group is almost planar at the N atom because of sp^2^-hybridization.^65^ The unit cell comprises eight molecules with two molecules in the asymmetric unit.

The key building block for assembling the crystal can be described as a *pseudo*-dimer (Figure 2b), as mentioned earlier. This species forms through alignment of the proline groups by way of two hydrogen bonds involving ester C=O groups and amide N–H groups. The respective O...H distances are 2.11(4) and 2.16(4) Å, while the closest contact between C atoms on adjacent proline rings is 4.45 Å. The BODIPY units appear as terminals to the proline-based spacer, exhibiting a B–B separation of 23.674 Å and a separation of 18.069 Å between the *meso*-C atoms. The *meso*-phenyl rings lie at dihedral angles of 93.7° and 99.1° with respect to the plane of the BODIPY unit. In the solid state, the amide group has the *trans* configuration, while the proline units reside in the *cis* geometry. An interesting feature of the *pseudo*-dimer is that the two *Boc* groups, and therefore the two ester groups, reside on the same side of an imaginary line connecting the two B atoms. The second B-P_1_ molecule has been rotated 180° about the long molecular axis.

**Figure 3:**
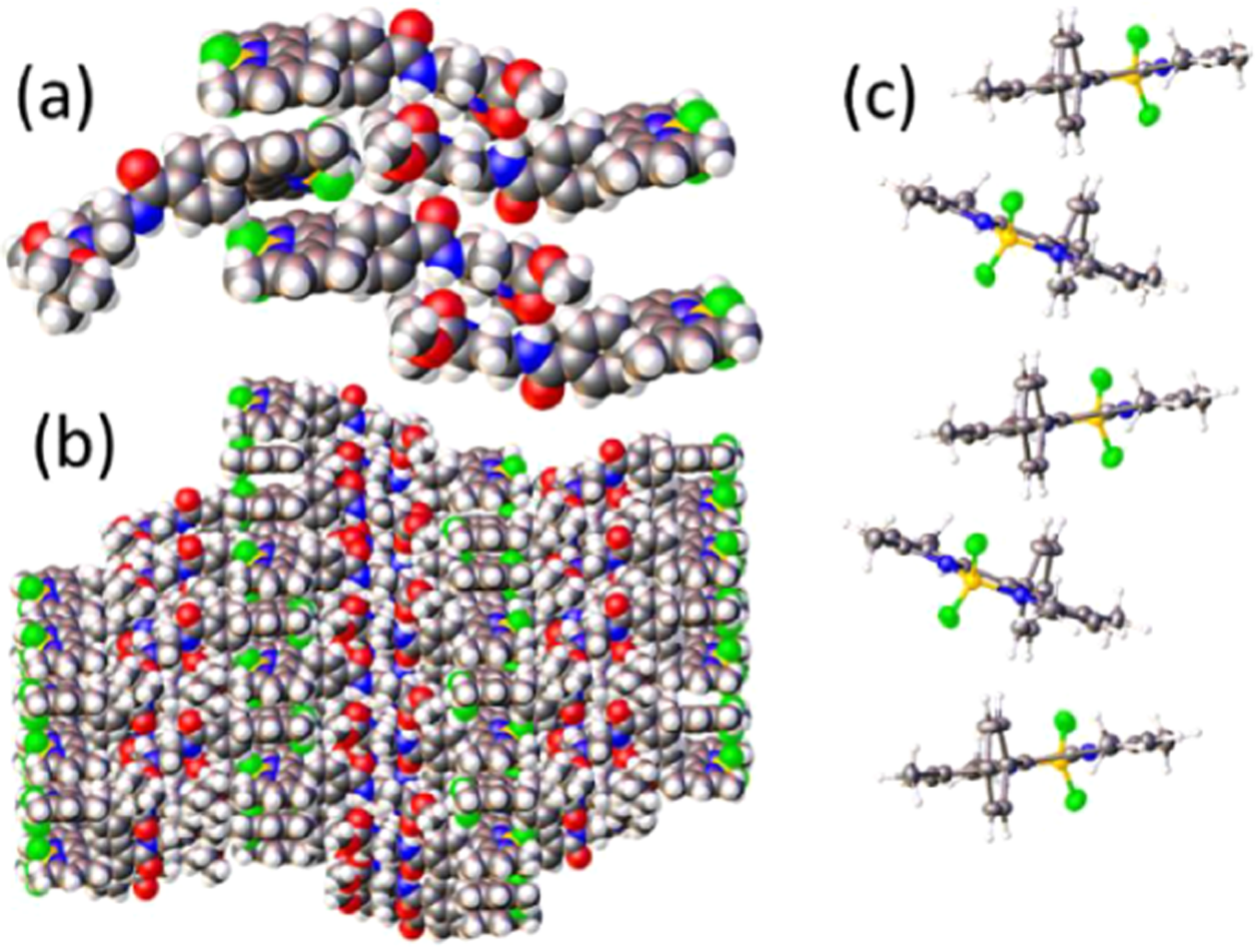
(a) Illustration of the packing of BODIPY residues to form a filament by intercalation (or interconnection). (b) Partial packing diagram showing the evolution of the filaments separated by stacked proline residues. (c) Arrangement of individual BODIPY chromophores within a single filament, where the proline residues have been omitted for clarity of presentation. Key: B, yellow; F, green; N, blue; and O, red.

The *pseudo*-dimer has translationally equivalent species located immediately above and below, with a B–B separation of ca. 9.915 Å but without hydrogen bonding between adjacent proline spacers (Figure 2b). Individual BODIPY residues within these layers are arranged in “stacked columns” with the *Boc* groups, and therefore the corresponding ester groups, positioned symmetrically on the same side. The spacing between neighboring BODIPY units is relatively large, but the planes defined by the BODIPY units are essentially colinear, the average interplane angle being ca. 1.3°. A second “column” intercalates into the first column, to give closer contacts between adjacent BODIPY units, but BODIPY-defined planes are tilted at ca. 32° (Figure 3a). The net result is a zig-zag staircase of closely spaced BODIPY units that resembles a “filament” of chromophores held in place by the aligned proline residues (Figure 3b). Figure 3c emphasizes the mutual arrangement of BODIPY chromophores within an emerging filament: note the alternation of the BF_2_ groups along the series.

**Figure 4:**
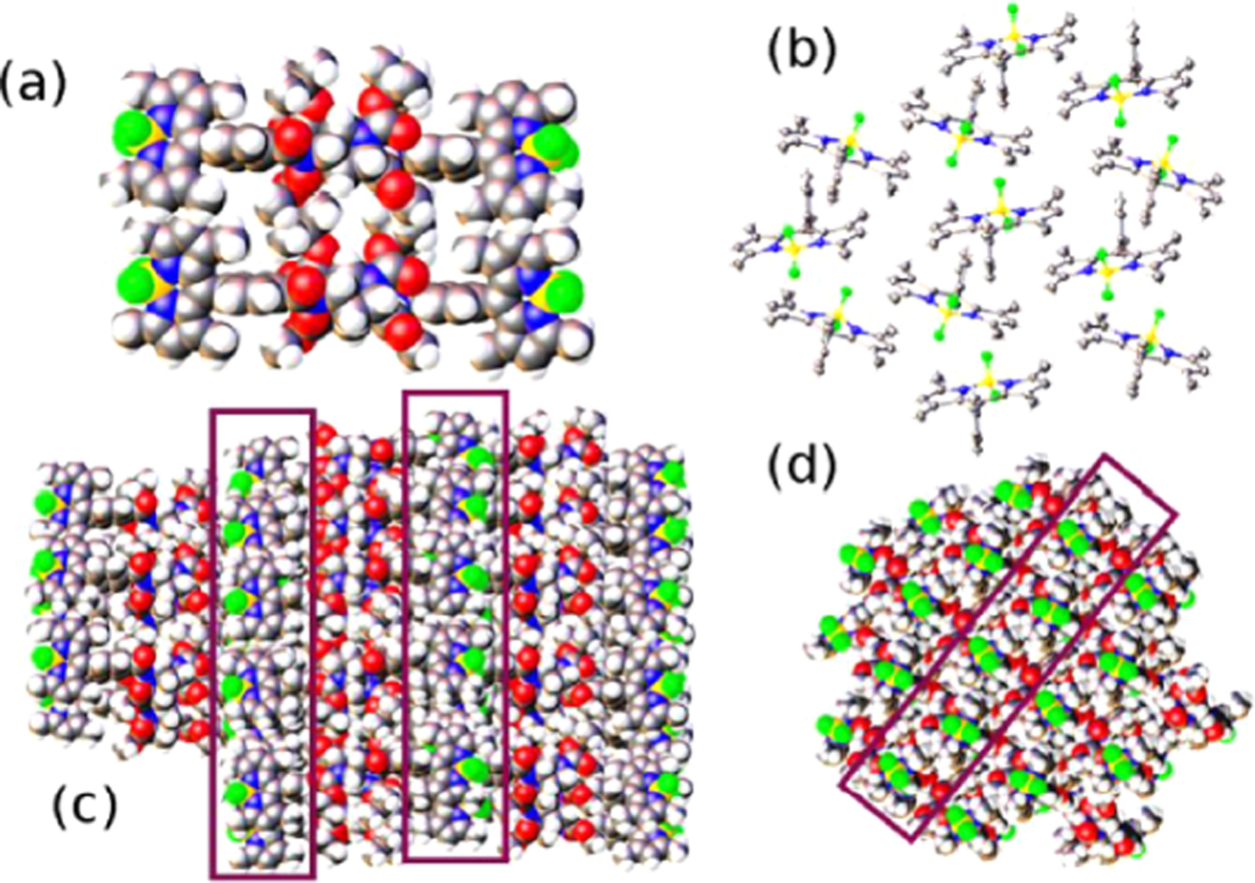
(a) Side-by-side assemblage of B-P_1_ species as the beginning of the evolution of the channels. (b) Partial packing of filaments illustrating the cofacial arrangement inherent to the filaments (running vertical) and the side-by-side arrangement used for the channels (running horizontal). (c) View of the crystal packing to illustrate the BODIPY-rich channels marked by two boxes. (d) Different views of the crystal packing to illustrate channel formation, as indicated by the box. Additional channels are apparent on either side of the box; The BF_2_ groups (yellow and green spheres) are good indicators of the structural pattern.

The 3D structure emerges by positioning filaments close together, with the B–B distances between neighboring BODIPYs being ca. 8.85 Å (Figure 4a). This leads to the construction of narrow (i.e., ca. 15 Å) channels packed with BODIPY residues, where adjacent chromophores are aligned side-by-side but the transition dipole moment vectors are not parallel. Figure 4b shows the close packing of filaments to form narrow channels containing BODIPY chromophores. These channels are isolated by proline residues (Figure 4c), with the overall effect being reminiscent of a lipid bilayer, where the proline groups mimic the hydrocarbon chains and BODIPY is the head group. The so-called channel adopts a zig-zag pattern at each level (Figure 4d). This structural motif is highly promising in terms of an effective artificial LHC since the filaments should possess a high absorption cross-section, but the narrow channels should help retain the exciton within that particular channel and thereby minimize losses due to exciton–exciton annihilation.

### Optical Properties of Crystalline B-P_1_

**Figure 5:**
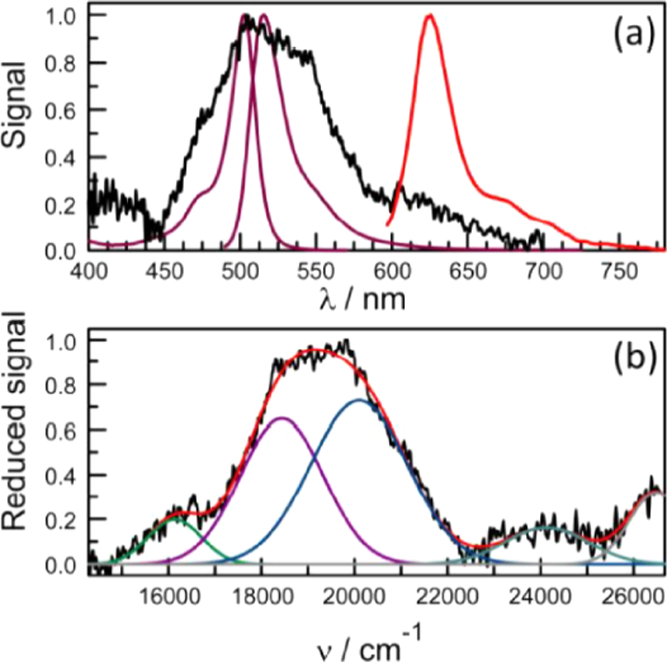
(a) Absorption (black curve) and fluorescence (red curve) spectra recorded for a single crystal of B-P_1_. Also shown (plum colored curves) are the corresponding spectra recorded in dilute solution. (b) Gaussian deconstruction of the reduced absorption spectrum for the crystalline sample. The black curve is the experimental spectrum, while the red curve is the simulated spectrum. Individual components refer to dimer (green curve), J-state (indigo curve), H-state (blue curve), and absorption to higher excited states (gray curves).

Absorption spectra recorded for crystals of B-P_1_ show broadened bands and additional transitions compared to the solution (Figure 5a). The spectra are subject to scattering effects and are both weak and noisy, despite signal averaging. Different samples gave identical absorption spectra, while the size of the crystal had no obvious effect on the spectral profile. Similar spectra were obtained by recording reflection and scattering signals rather than transmission (Figure S32). At first sight, the absorption spectrum for the crystal bears little resemblance to that for B-P_1_ in solution. Even so, the reduced absorption spectrum can be deconstructed into several regions in a logical manner that aids interpretation (Figure 5b). At long wavelength, for example, there is a weak, broad transition that has the hallmarks of an aggregate.^66^ There are several literature reports of dimers formed from BODIPY, where the red-shifted absorption spectrum appears in the far-red region.^67−69^ In our case, dimerization (or aggregation) might occur on the exterior of the crystal or in amorphous domains associated with the interior. The band maximum for this transition occurs at 618 nm. Higher-energy transitions can be resolved with maxima in the region of 415 and 370 nm and are assigned to a population of upper-lying excited singlet states.^70^

Additional absorption transitions are seen for the crystal, with maxima at 18,440 cm^–1^ (i.e., 542 nm) and 20,095 cm^–1^ (i.e., 498 nm). These transitions are relatively intense and, compared to solution, broaden considerably (Figure 5a): it is not possible to analyze this part of the spectrum as a single Gaussian component, but good fits are obtained using two components of equal half-width. We assign these latter absorption bands to Davydov splitting of the primary π,π* transition, with the H- and J-bands, respectively, lying at higher and lower energies.^71^ The mean energy of these two transitions is 19,270 cm^–1^, which should be compared with the excitation energy of isolated B-P_1_ in the solution (Δ*E* = 19,840 cm^–1^). This indicates that the crystal lattice provides a modest red shift relative to the solution. The extent of excitonic splitting (2*J*_D_) can now be established from the assigned peak positions as being 1,660 cm^–1^, while integration of the two absorption bands shows the ratio of oscillator strengths, which amounts to 1.3, lies in favor of the H transition. This level of excitonic coupling greatly exceeds that expected for interaction between the terminals of the *pseudo*-dimer but might be explained in terms of coupling between nearby BODIPY molecules within the channels. These molecules reside in proximity where it is understood that Kasha theory^59^ might not operate successfully. In contrast, the extended dipole method^61^ has been used to obtain coupling strengths for cyanine dyes embedded in 2D monolayers^62,72^ and for certain crystals.^73,74^ This latter approach can now be applied to B-P_1_ with the distance parameters being established by X-ray crystallography.

First, the excitonic coupling strength, *J*_D_, was computed for interaction between a randomly selected chromophore, with certain *x*, *y*, *z* coordinates, and nearby BODIPYs localized along the same filament (Figures S26 and S27). Thus, *J*_D_ can be determined from eq 1, where μ_TD_ refers to the transition dipole moment calculated for B-P_1_ in the solution, d is the length of the dipole moment vector, and (d) is the distance factor (see the Supporting Information). The effect of the solvent is included in the determination of the transition dipole moment, while the transition dipole length (*d*) is equated to the distance along the molecular axis from C_2_ to C_6_ (Figure S22 and Chart S1). The four intermolecular distances required to calculate the distance parameter are illustrated by way of Figure S23 and were measured from the crystal data for each pair of chromophores. It was found that values of *J*_D_ decrease rapidly with increasing separation (Figure S27), for example, *J*_D_ for interaction with BODIPY units immediately above and below the reference molecule account for 76% of the total *J*_D_ calculated for the nearest 10 chromophores sited along the filament.^62^ The second layer accounts for 15% of the total coupling along the filament, with the accumulated value adding up to 428 cm^–1^. The calculation was extended to include all chromophores lying along the filament within 25 Å of the reference molecule, but the net *J*_D_ falls well short of the experimental value (*J*_D_ = 830 cm^–1^). In particular, it should be emphasized that excitonic coupling to the nearest chromophores cannot account for the strong coupling derived from the analysis of the absorption spectrum.
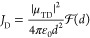
1
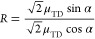
2The calculation was further expanded to include all BODIPYs lying within a 25 Å radius of the reference compound. This involved determining the distance factor for the two columns adjacent to the reference molecule (Figure S28). Again, a total of 10 BODIPYs was considered for each column, but the asymmetrical packing means that, unlike for the filaments, each pair of chromophores is subject to a different degree of excitonic interaction (Figure S29). In fact, out of the nearest 20 neighboring BODIPYs, only the closest four make a significant contribution to the intercolumn coupling. Thus, the total intercolumn exciton coupling energy is calculated to be 390 cm^–1^, of which ca. 70% is attributable to the closest four chromophores. The combined *J*_D_, derived for all 30 BODIPY residues lying within a 25 Å radius of the reference chromophore, can now be established as 820 cm^–1^, which is remarkably close to the experimental value of 830 cm^–1^. We consider this agreement to be fortuitous, but nonetheless, this finding supports the assignment made for the absorption spectrum. Although the strongest coupling (*J*_D_ = 326 cm^–1^) is with neighboring chromophores in the same filament, there is significant coupling (*J*_D_ = 288 cm^–1^) to the two adjacent columns (see Figure S30). This latter finding is taken as indication that the exciton will not be confined to a single filament.

It is common practice to use the ratio (*R*) of oscillator strengths for the H- and J-bands to estimate the average angle (α) between the transition dipole moment vectors according to eq 2.^75,76^ In our case, this expression leads to an average value for α of 53°. Because excitonic coupling occurs between pairs of chromophores distributed around the channel, it is not possible to compare this angle with an experimental measurement. Instead, we have calculated a mean angle for interaction between a reference BODIPY and the six neighboring chromophores responsible for the majority of the coupling energy (Figure S30). The mean angle so derived is ca. 50°, which adds further support for the validity of our model.

**Figure 6:**
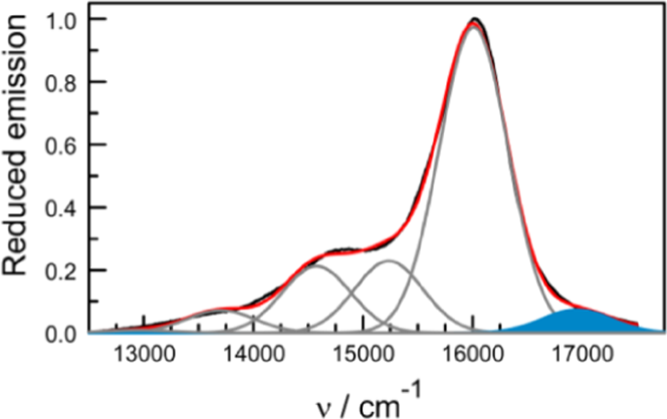
Reduced fluorescence spectrum recorded for single crystals of B-P_1_ with excitation at 470 nm. The experimental (black curve) and simulated (red curve) spectra are compared, while the individual Gaussian components are indicated as gray curves. The “hot” emission is highlighted as a solid blue component.

Excitation of single crystals of B-P_1_ at 470 nm gives rise to strong emission centered at 625 nm (Figure 6). The fluorescence lifetime, measured by time-correlated, single-photon counting, is 2.2 ± 0.2 ns, while the emission quantum yield is 0.34 ± 0.05. No fluorescence could be detected in the wavelength region around 520 nm, where we might expect to find emission from isolated BODIPY present as a surface contaminant or dislocation.^28−30^ It is noticeable, however, that the signal does not reduce to zero on the high energy side of the fluorescence profile, and there is a significant amount of hot fluorescence. This latter emission falls within the wavelength range, where the J-state might emit. The apparent peak of the reduced emission profile lies at 16,950 cm^–1^ (i.e., 590 nm) which, if the signal is attributable to the J-state, would correspond to a Stokes shift of 1,500 cm^–1^. The latter seems unreasonably high and, as a consequence, the hot emission is assigned to fluorescence from an upper vibronic level of the dimer responsible for the 625 nm emission (see below). Effectively, this assignment means that the J-state does not emit. That the J-state falls within the strong exciton coupling regime can be established^77^ by the fact that the magnitude of the free exciton bandwidth (*W* = 1,480 cm^–1^) far exceeds the reorganization energy for the monomer in the solution (*L*_B_ = 255 ± 10 cm^–1^). The former value was obtained from eq 3 on the assumption of a linear array of chromophores.^78^ This situation raises the possibility that the exciton is delocalized over several BODIPY units situated along the filament. Such delocalization is a common feature of J-aggregates^79−81^ and has been reported for several different classes of stacked chromophores.^82−85^ The absence of clear emission from the J-state, however, precludes further examination of this point.
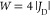
3
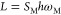
4
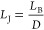
5

**Figure 7:**
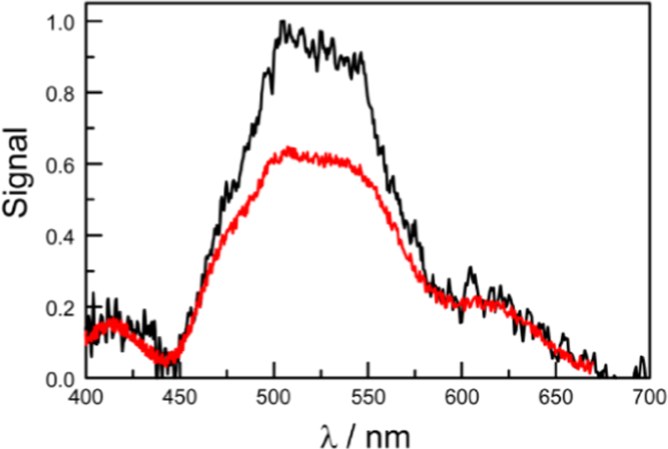
Comparison of absorption (black curve) and excitation (red curve) spectra recorded for a single crystal of B-P_1_. The reduced spectra were normalized across the 620–640 nm window. The emission wavelength used for the excitation spectrum was 700 nm.

Gaussian deconstruction^55^ of the reduced emission spectrum recorded for the crystal indicates that deactivation of the excited state involves a medium-frequency (*h*ω_M_ = 600 cm^–1^) vibronic mode (Figure 6). The energy of this coupled vibration is too high to represent intermolecular forces related to packing of the proline chains^86^ but is very close to that expected for an amide out-of-plane bending mode.^87^ The Huang–Rhys factor is 0.27 ± 0.02, while the FWHM has a value of 675 cm^–1^. From eq 4, the reorganization energy (*L*) associated with deactivation of the emissive state is 160 cm^–1^, which is much reduced relative to that for B-P_1_ in the solution (*L* = 255 cm^–1^). This indicates that the exciton is delocalized (i.e., *D* ≈ 2.5) over both BODIPY chromophores (eq 5). The fluorescence quantum yield measured for the crystals (Φ_F_ = 0.34 ± 0.05) is unusually high for a solid sample. Indeed, the radiative rate constant, *k*_RAD_, can be estimated as being ca. 1.6 × 10^8^ s^–1^, which is comparable to that found in the solution. This is a considerable increase when allowance is made for the mean emission energy (<ν_F_>).^88^ Indeed, the value of *k*_RAD_ expected for the crystal on the basis of eq 6 is 1.1 × 10^8^ s^–1^. This finding indicates a modest degree of super-radiance for the dimer.^89,90^
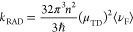
6The emission profile with a maximum at 625 nm is highly reproducible among different samples and is independent of crystal size. The latter is an important factor in eliminating self-absorption. An excitation spectrum recorded at an emission wavelength of 700 nm confirms that the fluorescence is associated with the absorption band having a maximum at 618 nm (Figure 7). This latter absorption feature has been attributed to an aggregated state^67−69^ since the band is reminiscent of spectral features common to noncovalently linked BODIPY dimers.^91,92^ Such dimers are often fluorescent, especially in cases where the transition dipole moment vectors are aligned parallel.^93^ The excitation spectrum also indicates that excitation into the split π,π* transition results in the same fluorescence spectral profile. There is but modest agreement between the excitation spectrum and the absorption spectrum over the wavelength range from 480 to 570 nm, however, despite good agreement around 620 nm. Most likely, self-absorption^94^ is responsible for the mismatch in the spectra since the absorbance of individual crystals is high over the cyan region. The net result is panchromatic absorption across most of the visible regions with very high light collection capacity by a single crystal. Incidentally, J-aggregates formed from π-extended BODIPY derivatives have been identified^95^ with absorption maxima (λ = 920 nm) at significantly lower energies.

The absence of fluorescence from either H- or J-states is a likely indication that trapping by the dimer is highly effective. This situation can be explained in terms of fast internal conversion from the H-state to the J-state, followed by EET from the J-state to the dimer (Figure S31).^96^ The observation of hot emission from the dimer is also consistent with EET from a high-energy donor. Even without self-absorption, the excitation spectrum shows that this trapping probability exceeds 75%. Such behavior seems inconsistent with random localization of dimer on the crystal exterior but could be explained in terms of dimer formation at the interface where the filaments become free from lattice control. In such a case, the dimer would be a logical terminus for the filament and would be an integral part of each BODIPY stack. As such, it would be ideally positioned to act as a trap^97^ for excitons migrating randomly within a particular channel.

Although this aggregated state plays a crucial role in establishing the light-harvesting character of B-P_1_, there is a complete scarcity of structural information for this species. The observation that the emitting state is delocalized over a pair of chromophores indicates close positioning of the respective BODIPY molecules. The large red shift between absorption maxima for monomer and dimer, which amounts to 3,700 cm^–1^, is consistent with an in-line arrangement of the transition dipole moment vectors.^59,60^ In addition, the unusually small Stokes shift observed for the dimer implies a fairly rigid geometry that does not change on excitation. These properties appear fully consistent with the side-by-side structural motif highlighted in Figure 4a and should be easily accommodated at the crystal surface.

## Concluding Remarks

We have presented an account of events that might follow illumination of a crystalline sample of B-P_1_, and these should be considered in terms of an artificial LHC. Self-association, facilitated by hydrogen bonding, of proline units^64,83^ into a *pseudo*-dimer is considered to be a key step in crystal packing. The latter favors establishment of filaments rich in BODIPY chromophore which, in turn, promotes excitonic coupling^78^ between nearby BODIPY residues. Interestingly, the lattice packing splits the absorption band into two components of almost equal intensity and this is crucial for the development of panchromatic antennae. Most self-assembling systems result in the formation of H- or J-stacks having fairly narrow absorption bands, but this is not conducive for effective light collection.^81^ Exciton delocalization^85^ cannot be ruled out for the J-state, but excitonic coupling is not restricted to a parallel filament. The probability of placing two or more excitons on a single filament is low at all reasonable excitation levels such that exciton–exciton annihilation should be relatively unimportant.

One of the most interesting features of these crystals relates to what appears to be the capping of the filaments with a BODIPY-based dimer that emits red fluorescence. The dimer, which appears to be mildly super-radiant,^94,95^ can be observed by absorption spectroscopy and therefore must be present at a reasonable concentration. Given that the crystal packing appears highly suited for dimer formation and that exciton trapping by the dimer is extremely efficient, we speculate that the dimer predominates at the interface and is an integral part of the channels. If correct, this unique pattern means that a high proportion of excitons created inside the crystal will be emitted at the surface.^96^ Such properties are highly desirous for an artificial LHC.^97−101^

Indeed, the special optical and transport properties inherent to molecular aggregates have long been recognized and utilized for a variety of applications. At the dawn of photography, for example, it was realized that the active sensitizers for color films were aggregates of cyanine dyes.^102^ More recently, self-assembled flexible fluorescent fibers have been developed that look promising in terms of thin-film optoelectronic devices.^103,104^ With particular significance to our work, we note that the large absorption cross-section of certain molecular aggregates, together with rapid exciton migration, has been applied to enhance fluorescence from a very low concentration of embedded dye^105^ or dye adhered to the exterior.^106,107^ Such behavior could be applied to sensitize certain types of organic solar cells.^108,109^ We have found that crystals of B-P_1_ function as efficient sensitizers for sulfonated aluminum phthalocyanine deposits on the surface, at concentrations where the dopant cannot be detected by absorption spectroscopy and its fluorescence is barely visible on direct excitation. In this case, the dimer acts as an intermediary to transfer excitation energy from the filament to the dopant in much the same way that the Fenna–Mathews–Olsen complex couples natural LHCs to their reaction center.^110^

## Supporting Information Available

Compound characterization, tabulated data for the crystal structure, computed and experimental molecular structures, details for the various spectroscopic calculations, and additional photophysical information (PDF)
