# Supplementary material for: Light-Harvesting Crystals Formed from BODIPY-Proline Biohybrid Conjugates: Antenna Effects and Excitonic Coupling
Source: J Phys Chem A. 2022 Mar 1;126(9):1530–41. doi: 10.1021/acs.jpca.2c00035 (PMC9097531; doi:10.1021/acs.jpca.2c00035)
Supplement: Supplementary file 1 — jp2c00035_si_001.pdf [file jp2c00035_si_001.pdf]

Light-Harvesting Crystals Formed from BODIPY-Proline Bio-Hybrid Conjugates: Antenna Effects and Excitonic Coupling

Sara M. Waly,[a] Joshua K. G. Karlsson,[a] Paul G. Waddell,[b] Andrew C. Benniston,[b,*] and Anthony Harriman[a,*]

(a) Molecular Photonics Laboratory and (b) Crystallography Laboratory, School of Natural & Environmental Sciences, Bedson Building, Newcastle University, Newcastle upon Tyne, NE1 7RU, UK

SUPPORTING INFORMATION

Total number of pages … 45

Table of Contents:

S1. Synthesis and compound characterization S2

S2. X-ray crystallography and quantum chemical calculation S15

S3. Spectroscopy in liquid solution S32

S4. Reflectance spectrum recorded for crystalline B-P_1 S45

S1. Synthesis and compound characterization

Material and methods

All chemicals and reagents were purchased from commercial suppliers and were used as received without further purification. All reactions were performed in dried glassware under an atmosphere of nitrogen using Schlenk techniques. Dichloromethane was distilled over calcium hydride. ^{1}H, $^{13}C\{^{1}H\}$, $^{19}F\{^{1}H\}$, $^{11}B\{^{1}H\}$ NMR spectra and 2D NMR spectra were recorded on JEOL ECS 400, Bruker Avance 300, Jeol Lambda 500, or Bruker Avance 700 MHz instruments and referenced to the residual proton signals of the solvent. Mass spectrometry analysis was performed using Electrospray Ionization and obtained from the EPSRC-sponsored Mass Spectrometry Service at Swansea Medical School.

Preparation of 1

2,4-Dimethylpyrrole (570 mg, 22 mmol, 2 eq.) and methyl-4-formylbenzoate (492 mg, 11 mmol, 1 eq.) were dissolved in anhydrous CH_2Cl_2 (225 mL) and the mixture was degassed by bubbling with dried nitrogen gas for 30 min. Two drops of trifluoroacetic acid (TFA) were added to the reaction mixture, which was stirred overnight at room temperature under a nitrogen atmosphere. After disappearance of the aldehyde (as monitored using thin-layer chromatography, TLC), a solution of 2,3-dichloro-5,6-dicyano-p-benzoquinone (DDQ) (2.5 g, 11 mmol, 1 eq.) in a mixture of anhydrous CH_2Cl_2 (25 mL) and anhydrous THF (25 mL) was added dropwise over a period of 30 min to the mixture before being stirred for a further 4 h at room temperature. The reaction mixture was cooled in an ice bath, then N,N-diisopropylethylamine (8 mL) was added to the reaction mixture and after stirring for 15 min, $BF_3.OEt_2$ (9 mL) was added dropwise and the mixture was stirred at room temperature overnight. The reaction mixture was quenched with cold water (500 mL) and saturated aqueous $NaHCO_3$ was added and extracted by CH_2Cl_2 (3 × 200 mL). The combined organic layers were washed with brine and dried over Na_2SO_4 before being filtered. The solvent was removed under reduced pressure. The crude product was purified using column chromatography on silica gel (CH_2Cl_2/PE, 2/1) to afford shiny, brick-red crystals of compound **1** (1.3 g, 3.4 mmol, 80%). R_F: 0.73 (DCM). Mp: 115-116 ^{o}C. ^{1}H NMR (300 MHz, Chloroform-d) δ = 1.29 (s, 6H, $2CH_3$), 2.49 (s, 6H, $2CH_3$), 3.90 (s, 3H,-$COOCH_3$), 5.92 (s, 2H, β-pyrrole), 7.34 (d, J = 8.2 Hz, 2H, ArH), 8.11 (d, J = 8.2 Hz, 2H, ArH); ^{13}C NMR (75 MHz, Chloroform-d) δ = 14.5, 14.6, 52.3, 121.4, 128.3, 130.3, 130.8, 130.9, 140.2, 142.8, 155.9 and 166.4. ^{11}B NMR δ =

(96 MHz, Chloroform-d) δ 0.74 (t, JB-F = 32.2 Hz). ^{19}F NMR (282 MHz, Chloroform-d) δ = -146.14 (q, JF-B = 32.2 Hz).

*Preparation of **2***

Compound **1** (38.2 mg, 0.1 mmol, 1 eq.) was dissolved in $CH_2Cl_2/C_2H_5OH/H_2O$ (30/30/5) and NaOH (200 mg, 5 mmol, 50 eq) was added. The reaction mixture was refluxed for 4 hours before the mixture was acidified using (0.1 M HCl) until pH = 3. The product was extracted by CH_2Cl_2 (3 × 100 mL) and washed with brine before being dried over anhydrous Na_2SO_4. The solution was filtered and concentrated under reduced pressure to give red crystals of compound **2** (33.1 mg, 95%, 0.09 mmol). R_F: 0.67 (CH_2Cl_2/CH_3OH = 4/1). Mp: 117-118 °C. $_1H$ NMR (400 MHz, Chloroform-d) δ = 1.40 (s, 6H, $2CH_3$), 2.59 (s, 6H, $2CH_3$), 6.02 (s, 2H, β-pyrrole), 7.47-7.48 (d, J = 8.0 Hz, 2H, Ar-H), 8.27-8.28 (d, J = 8.0 Hz, 2H, Ar-H). ^{13}C NMR (176 MHz, Chloroform-d): δ = 14.5, 14.6, 121.5, 128.6, 129.73, 130.8, 130.9, 139.9, 140.7, 142.8, 143.1, 156.1 and 169.6. ^{11}B NMR (96 MHz, Chloroform-d) δ = 0.74 (t, JB-F = 32.2 Hz). ^{19}F NMR (282 MHz, Chloroform-d) δ = -146.02 (q, JF-B = 32.2 Hz).

Preparation of B-P_1

To a 50 mL Schlenk flask, under a nitrogen atmosphere, compound **2** (0.45 g, 1.22 mmol, 1 eq.) was dissolved in dry DMF (20 mL). DCC (0.52 g, 2.5 mmol, 2 eq.) was added, followed by HOBt (0.33 g, 2.45 mmol, 2 eq.). The reaction mixture was stirred for 1 h at room temperature. The amine **3** (0.34 g, 1.22 mmol, 1 eq.) was added and the reaction mixture was stirred at room temperature for 12 h. The precipitate was removed by filtration and then the filtrate was concentrated under vacuum. The resulting residue was purified by silica gel column chromatography using CH_2Cl_2/CH_3OH (3/1). The compound B-P_1 was isolated as an orange solid (0.54 g, 75%, 0.9 mmol), R_F: 0.87 (CH_2Cl_2/CH_3OH, 4/1). Mp: 120-121 °C. ^1H NMR (400 MHz, Methanol-d) δ = 1.30 (s, 6H, $2CH_3$), 1.37 (2 s, 9H, CH_3 (Boc), rotamers), 2.03-2.05 (m, 1H, H-cyclopentane), 2.48 (s, 6H, 2 CH_3), 2.57-2.63 (m, 1H, H-cyclopentane), 3.59-3.67 (m, 2H, H-cyclopentane), 3.72 (s, 3H, OCH_3), 4.28- 4.40 (m, 1H, H-cyclopentane), 4.83-4.86 (m, 1H, H-cyclopentane), 5.92 (s, 2H, β-pyrrole), 7.32-7.35 (d, J = 7.90 Hz, 2H, ArH), 7.75-7.78 (br, 1H, NH), 7.89-7.91(d, J =7.90 Hz, 2H, ArH). ^{13}C-NMR (75.5 MHz, Chloroform-d): δ = 14.5, 28.2, 29.6, 30.3, 35.7, 36.6, 48.3, 49.4, 52.6, 52.8, 53.4, 53.9, 57.8, 80.8, 121.4, 127.8, 128.5, 131, 134.4, 138.4, 140.4, 143, 153.5, 154.3, 155.9, 165.2 and 175.7. ^{11}B NMR (96 MHz, Chloroform-d) δ = 0.75 (t, JB-F = 32.2 Hz). ^{19}F NMR (282 MHz, Chloroform-d) δ = -146.02 (q, JF-B = 32.2 Hz).

HRMS: (ASAP+) calcd. for $C_{31}H_{39}BF_2N_4O_5$ $[M]^+$: 594.28, found $[M]^+$ 594.28, $[M+H]^+$ 595.28, $[M+Na]^+$ 617.27, $[M-F]^+$ 575.28.

Synthesis of B-P₁-NH

Compound B-P_1 (16 mg, 0.02 mmol, 1 eq.) was treated with 4M HCl in 1,4-dioxane (1 mL, 0.02 mmol, 1 eq.) and the reaction stirred overnight at room temperature. The volatiles were evaporated under reduced pressure to give a dark reddish solid. Saturated $NaHCO_3$ was added to neutralize the solution. Then CH_2Cl_2 was added and the organic layer was separated and concentrated to afford the desired product B-P_1-NH as an orange solid (14 mg, 88%, 0.017 mmol). R_F: 0.31 (CH_2Cl_2/CH_3OH = 4/1). Mp: 119-120 °C. ^{1}H NMR (300 MHz, Methanol-d) δ = 1.20 (s, 6H, $2CH_3$), 1.93-1.96 (m, 1H, H-cyclopentane), 2.29-2.33 (m, 1H, H-cyclopentane), 2.40 (s, 6H, $2CH_3$), 2.50-2.52 (m, 1H, H-cyclopentane), 3.05-3.11 (m, 2H, H-cyclopentane), 3.60 (s, 3H, OCH_3), 3.82-3.83 (m, 1H, H-cyclopentane), 4.60-4.62 (br, 1H, NH), = 5.83 (s, 2H, β-pyrrole), 7.22-7.24 (d, 2H, J = 7.80 Hz, ArH), 7.26-7.28 (br, 1H, NH), 7.80-7.82 (d, 2H, J = 7.80 Hz, ArH). ^{13}C-NMR (75.5 MHz, Methanol-d): δ = 13.1, 13.5, 29.5, 35.4, 50.9, 51.2, 51.5, 58.3, 121, 128, 128.3, 130.8, 134.9, 138.3, 140.8, 142.9, 159, 167.81, 167.83, 174. ^{11}B NMR (96 MHz, Methanol-d): δ = 0.73 (t, JB-F = 32.2 Hz). ^{19}F NMR (282 MHz, Methanol-d) δ = -146.18 (q, JF-B = 32.2 Hz). HRMS: (ASAP$^+$) calcd. for $C_{26}H_{31}BF_2N_4O_3$ $[M]^+$: 494.25, found $[M]^+$ 494.23, $[M+H]^+$ 495.23 , $[M+Na]^+$ 517.21.

Synthesis of B-P₁-COOH

Compound B-P_1 (50 mg, 0.085 mmol, 1 eq.) was dissolved in $CH_2Cl_2/C_2H_5OH/H_2O$ (30/30/5) before addition of sodium hydroxide (200 mg, 50 eq.). The mixture was refluxed for 4 h and after cooling the solution was neutralized using 0.1 M HCl before being extracted with CH_2Cl_2 (5×30 mL). The organic phase was washed with brine and water and dried over anhydrous Na_2SO_4 to provide the crude product. This material was used without further purification to give B-P_1-COOH as an orange solid (48.50 mg, 97%, 0.083 mmol). R_F: 0.35 (CH_2Cl_2/CH_3OH, 7/3). Mp: 122-124 °C. ^{1}H NMR (700 MHz, Chloroform-d) δ = 1.35 (s, 6H, $2CH_3$), 1.50 (2 s, 9H, $3CH_3$ (Boc, rotamers), 2.33-2.43 (m, 1H, H-cyclopentane), 2.55 (s, 6H, $2CH_3$), 2.61-2.65 (m, 1H, H-cyclopentane), 3.66-3.67 (m, 2H, H-cyclopentane), 4.59-4.62 (m, 1H, H-cyclopentane), 4.69-4.72 (m, 1H, H-cyclopentane), 5.98 (s, 2H, β-pyrrole), 7.37-7.40 (d, J = 7.90 Hz, 2H, ArH), 7.59 (br, 1H, NH), 7.95-7.97 (d, J = 7.90 Hz, 2H, ArH). ^{13}C-NMR (75.5 MHz, Methanol-d): δ = 15.6, 17.9, 29.3, 31.3, 36.8, 38.2, 51.2, 54.24, 55.2, 58.5, 82.2. 123, 130, 132,7, 133.4, 136.4, 140.2, 142.7, 144.4, 144.8, 156.8, 157.6, 157.7, 168.3. ^{11}B NMR (96 MHz, Chloroform-d) δ = 0.70 (t,

JB-F = 32.2 Hz). ^{19}F NMR (282 MHz, Chloroform-d) δ = -146.3 (q, JF-B = 32.2 Hz). HRMS: (ASAP$^+$) calcd. for $C_{30}H_{35}BF_2N_4O_5$ [M]$^+$: 580.27, found [M]$^+$ 580.27, [M+H]$^+$ 581.27.

Preparation of B-P$_2$-B

To a 50 mL Schlenk flask, under a nitrogen atmosphere, compound B-P$_1$-COOH (30 mg, 0.1 mmol, 1 eq.) was dissolved in dry DMF (5 mL). The solution was stirred for 10 mins under nitrogen before addition of N,N-diisopropylethylamine (3 μL, 0.2 mmol, 2 eq.), followed by COMU (56 mg, 0.2 mmol, 2 eq.). The reaction mixture was stirred for 5 mins before adding B-P$_1$-NH (49 mg, 0.1 mmol, 1 eq.). The reaction mixture was stirred at room temperature for a further 12 h. The solvent was removed by distillation, then the crude product was dissolved in dichloromethane and washed successively with 0.1 M HCl, $NaHCO_3$, brine and water. The solvent was evaporated under vacuum. The residue was purified by silica gel column chromatography using CH_2Cl_2/CH_3OH (7/3). Compound B-P$_2$-B was isolated as an orange solid (70 mg, 71%, 0.06 mmol), R_F: 0.67 (CH_2Cl_2/CH_3OH 4/1). Mp: 122-124°C. ^{1}H NMR (500 MHz, Chloroform-d) δ = 1.25 (s, 6H, 2CH_3), 1.34 (s, 3H, CH_3), 1.45 (2 s, 9H, CH_3 (Boc), rotamers), 2.12-2.17 (m, 1H, H-cyclopentane), 2.55 (s, 12H, 4CH_3), 2.96-2.99 (m, 2H, H-cyclopentane), 3.74 (s, 3H, OCH_3), 3.78-3.85 (m, 3H, H-cyclopentane), 4.20-4.23 (m, 1H, H-cyclopentane), 4.37 (m, 1H, H-cyclopentane), 4.56-4.62 (m, 1H, H-cyclopentane), 4.72 (m, 1H, H-cyclopentane), 5.03-5.10 (m, 2H, H-cyclopentane), 5.98 (s, 4H, β-pyrrole), 7.34-7.40 (d, J = 7.90 Hz, 4H, ArH), 7.69-7.94 (m, 1H, NH), 7.93-7.98 (d, J = 7.90 Hz, 4H, ArH), 8.05-8.07 (m, 1H, NH). ^{13}C-NMR (75.5 MHz, Chloroform-d) δ = 14.1, 14.6, 28.4, 29.7, 30.3, 31.5, 34.2, 49.1, 49.9, 52.8, 53.4, 54.6, 54.9, 56.9, 58.4, 80.7, 121.4, 125.5, 127.7, 128, 128.2, 128.7, 130.9, 131, 133.9, 134.6, 135.7, 138.3, 138.8, 140, 140.4, 142.7, 142.8, 151.5, 154.3, 155.9, 164.7, 165.2, 173.4, 175. ^{11}B NMR (96 MHz, Chloroform-d) δ = 0.78 (t, JB-F = 32.8 Hz). ^{19}F NMR (282 MHz, Chloroform-d) δ = -146.11 (q, JF-B = 32.2 Hz). HRMS: (ASAP$^+$) calcd. for $C_{56}H_{62}B_2F_4N_8O_7$ [M]$^+$: 1056.49, found [M]$^+$ 1056.49, [M+Na]$^+$ 1079.47, [M-F]$^+$ 1037.48, [M+NH_4]$^+$ 1074.52.

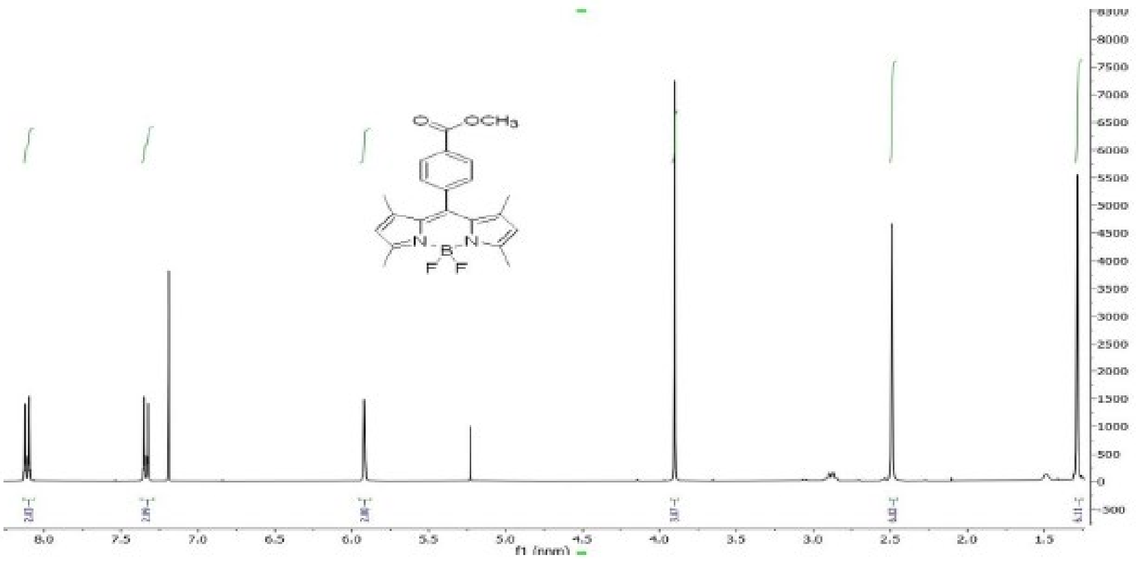

Figure S1. ^{1}H NMR (300 MHz, $CDCl_3$) spectrum recorded for compound **1**.

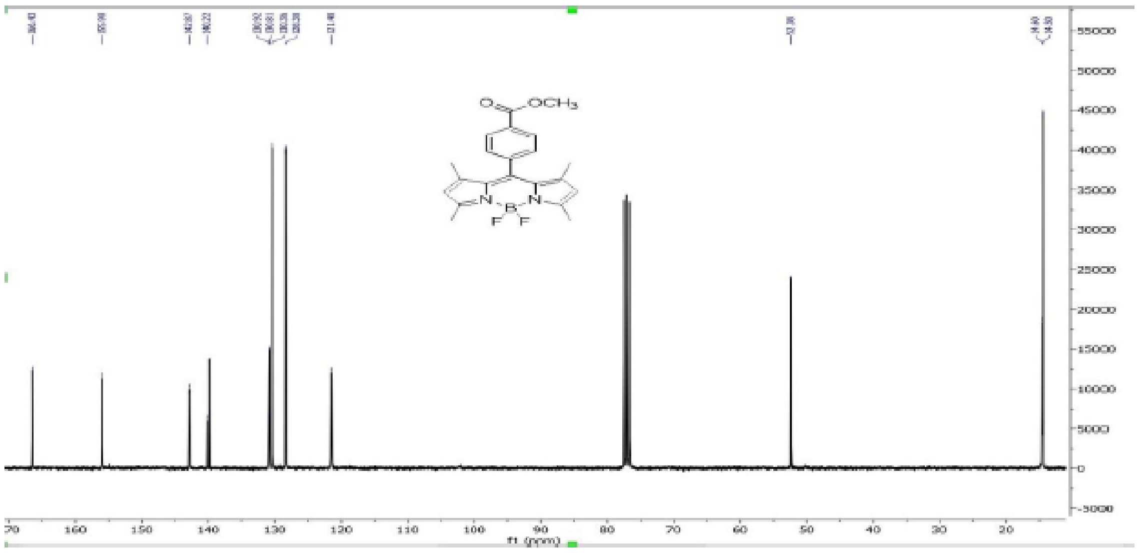

Figure S2. ^{13}C NMR (75 MHz, $CDCl_3$) spectrum recorded for compound **1**.

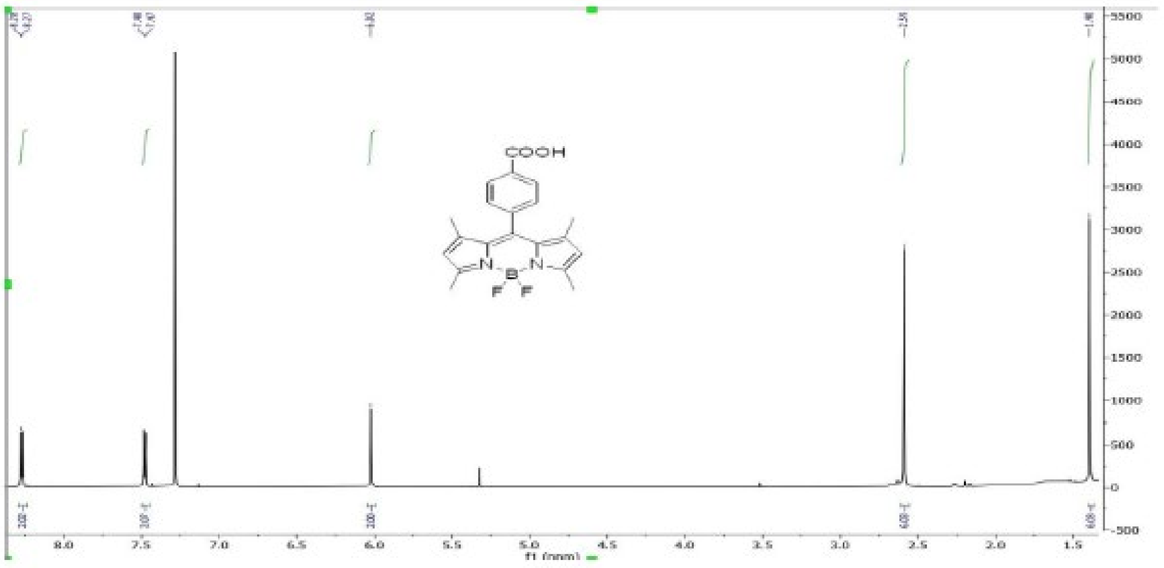

Figure S3. ^{1}H NMR (300 MHz, $CDCl_3$) spectrum recorded for compound **2**.

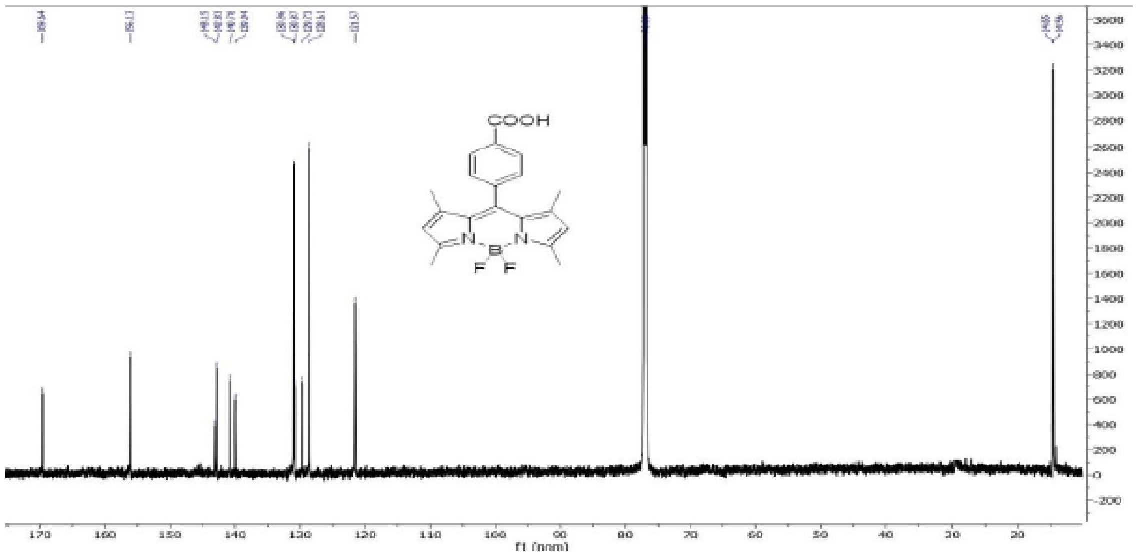

Figure S4. ^{13}C NMR (75 MHz, $CDCl_3$) spectrum recorded for compound **2**.

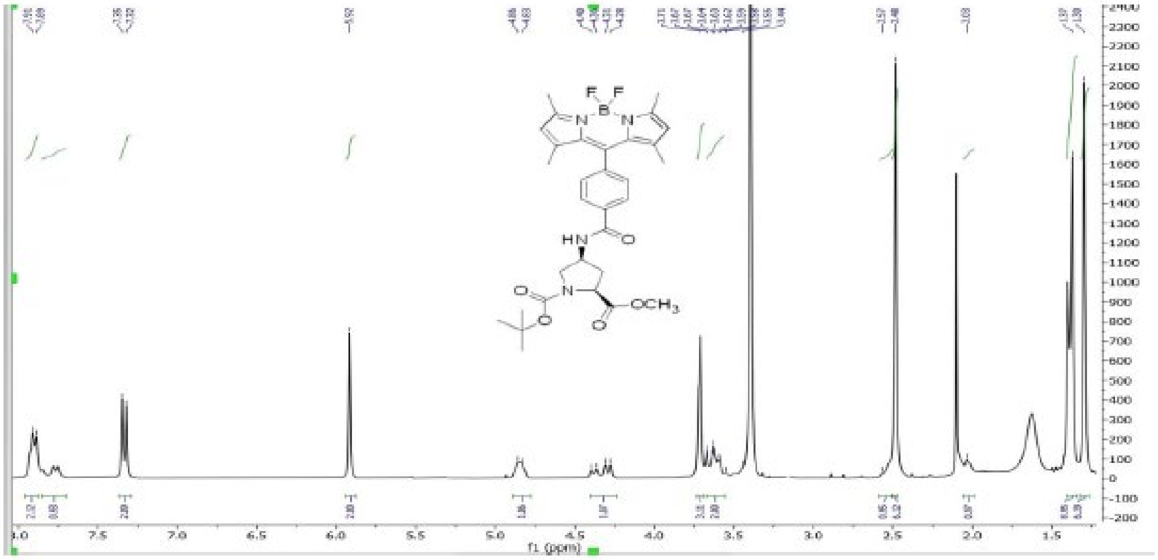

Figure S5. ^{1}H NMR (400 MHz, CD_3OD) spectrum recorded for compound **B-P$_1$**.

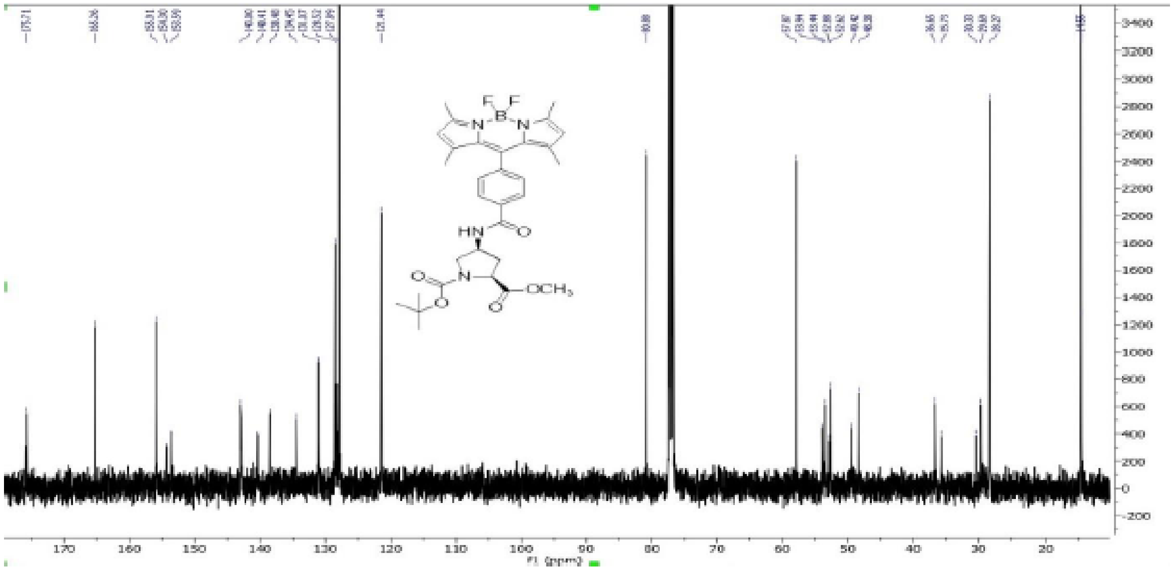

Figure S6. ^{13}C NMR (75 MHz, $CDCl_3$) spectrum recorded for compound **B-P$_1$**.

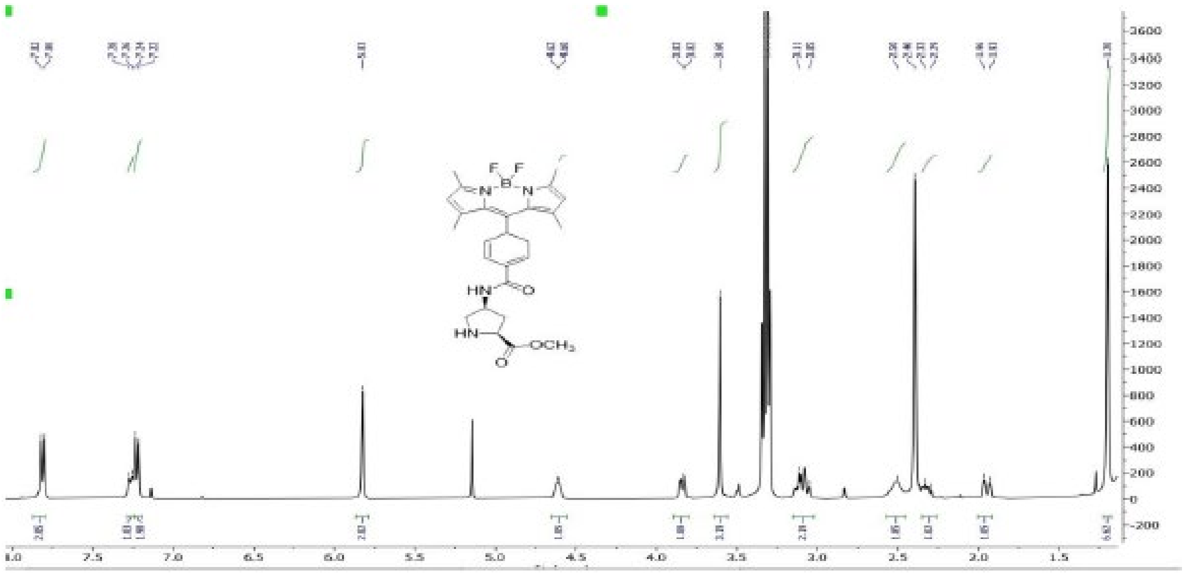

Figure S7. ^{1}H NMR (300 MHz, CD_3OD) spectrum recorded for compound **B-P$_1$-NH**.

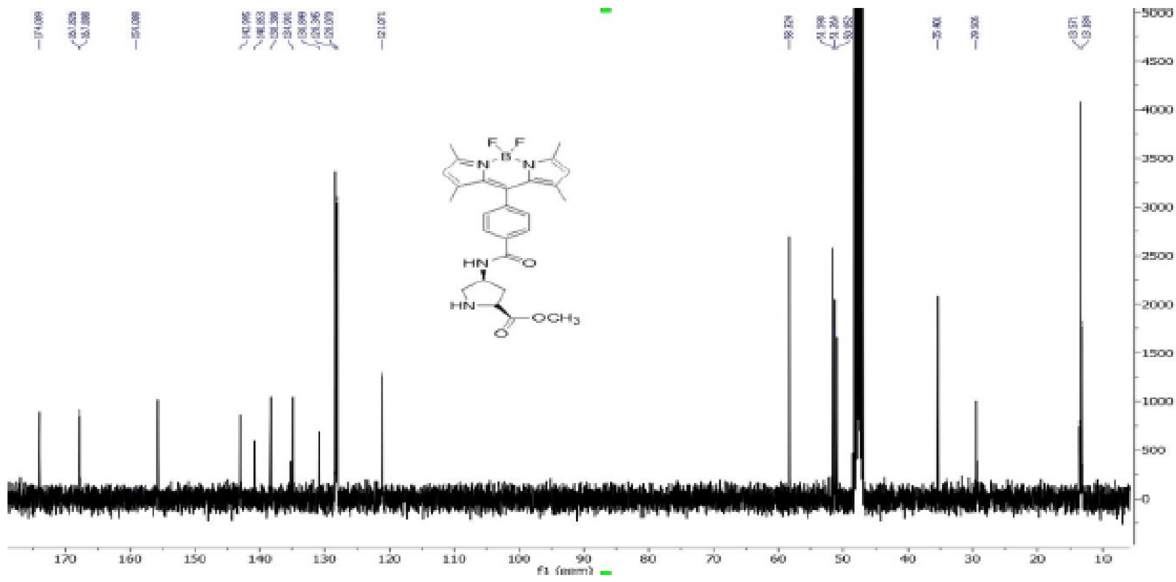

Figure S8. ^{13}C NMR (75 MHz, $CDCl_3$) spectrum recorded for compound **B-P$_1$-NH**.

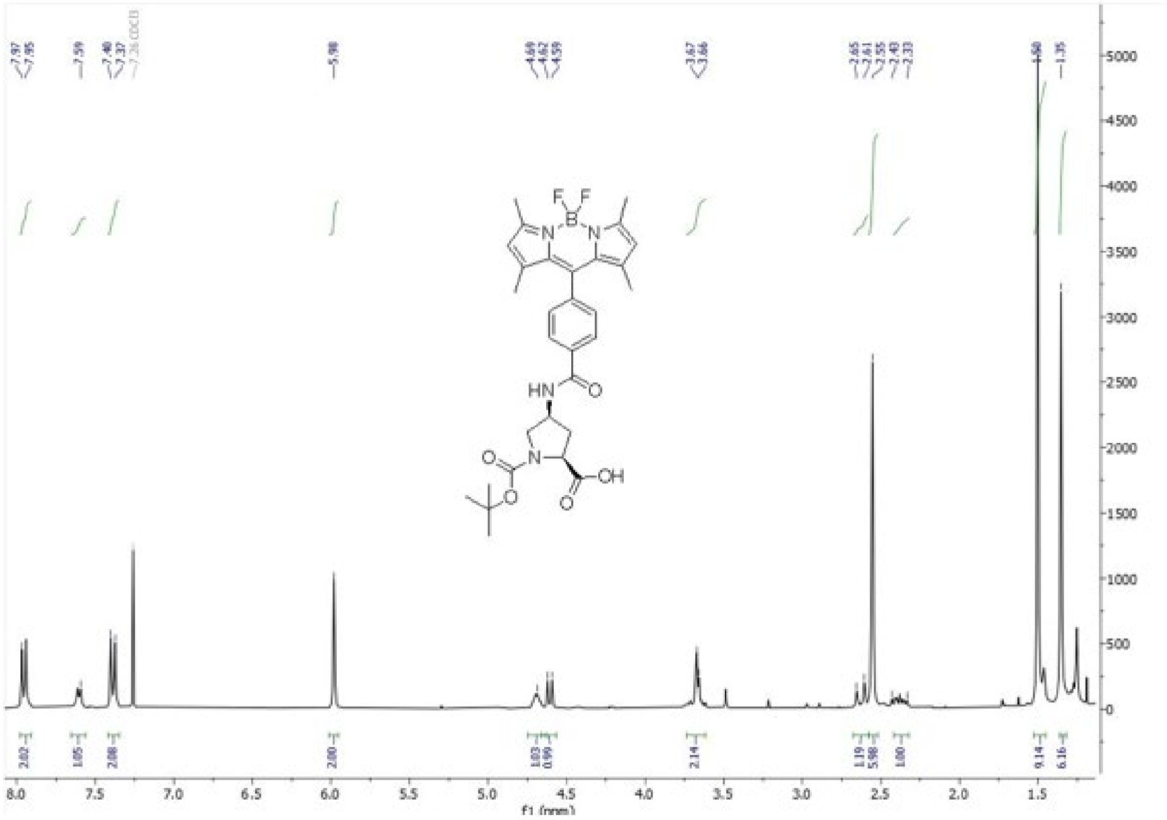

Figure S9. ^{1}H NMR (700 MHz, $CDCl_3$) spectrum recorded for compound **B-P$_1$-COOH**.

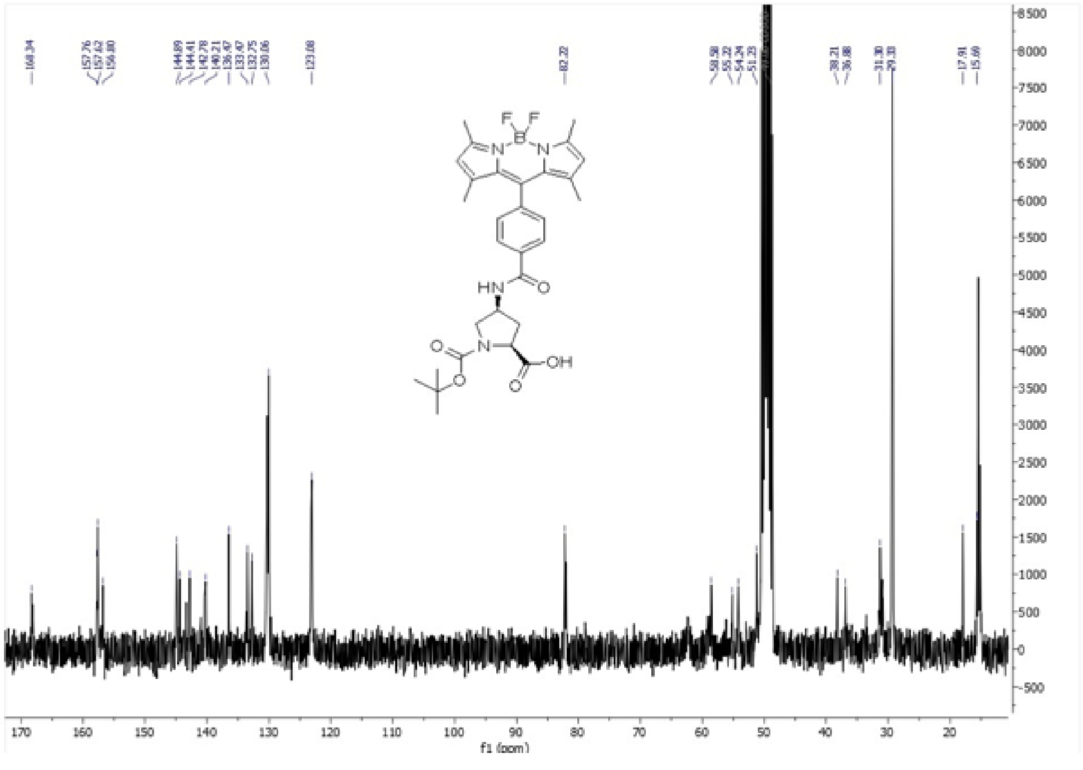

Figure S10. ^{13}C NMR (700 MHz, CD_3OD) spectrum recorded for compound **B-P$_1$-COOH**.

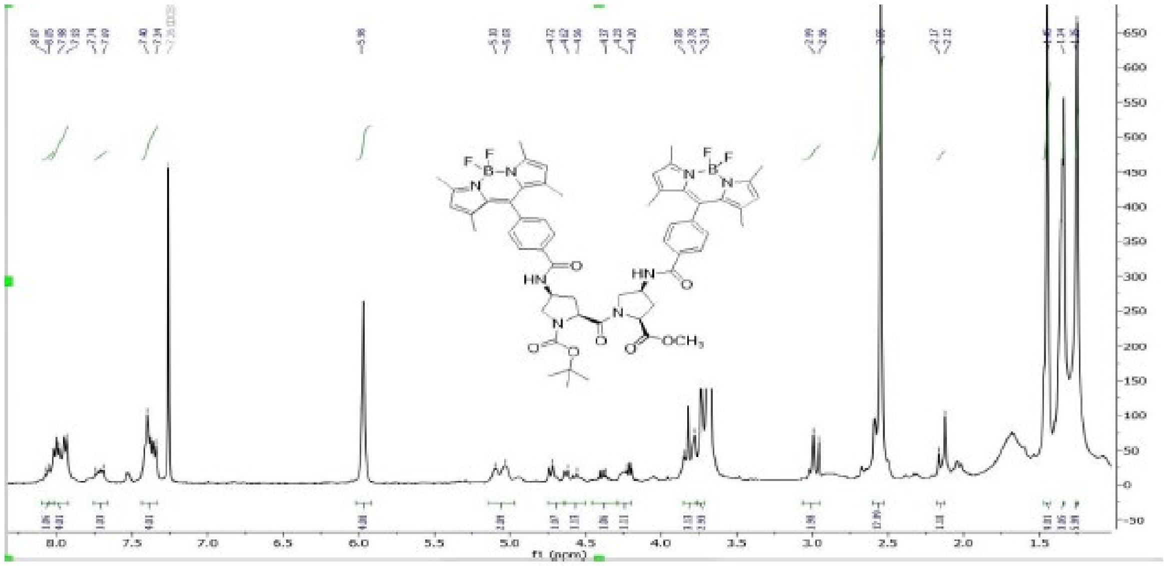

Figure S11. ^{1}H NMR (500 MHz, $CDCl_3$) spectrum recorded for compound **B-P$_2$-B**.

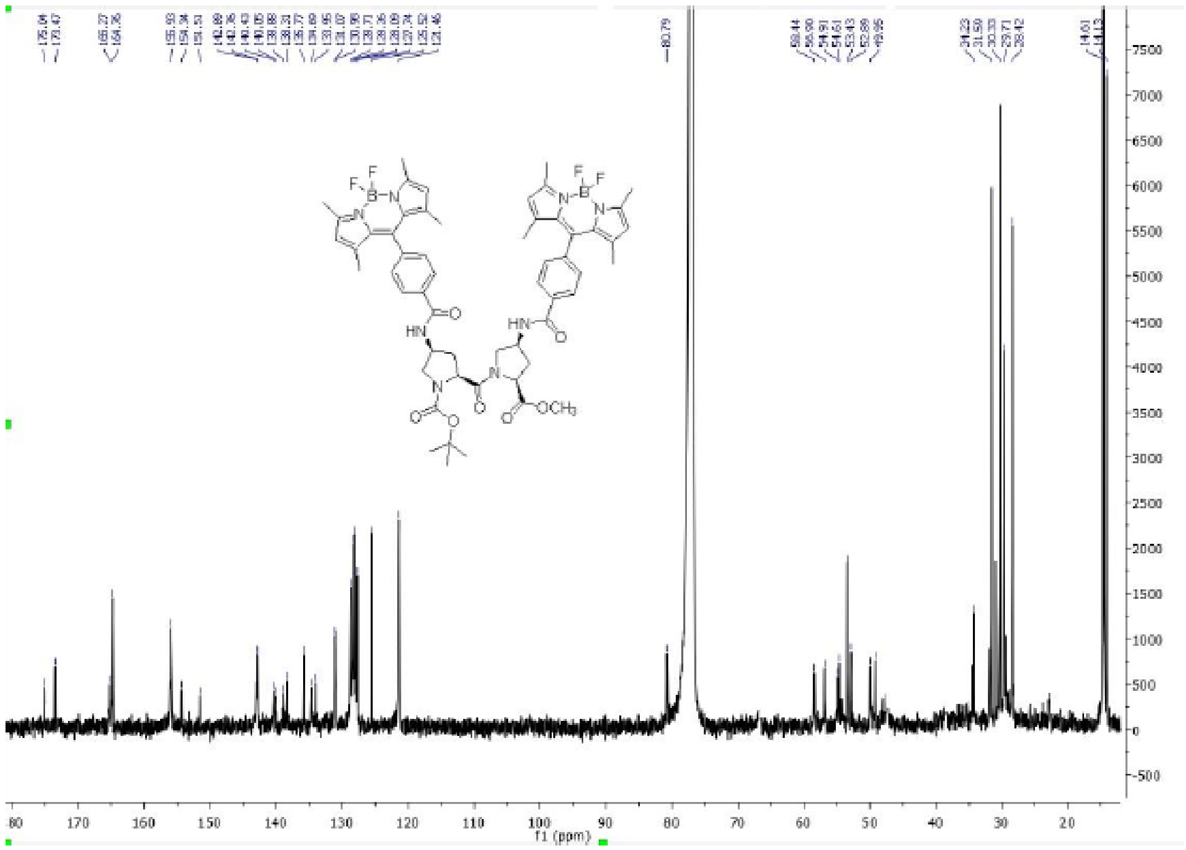

Figure S12. ^{13}C NMR (75 MHz, $CDCl_3$) spectrum recorded for compound **B-P$_1$-B**.

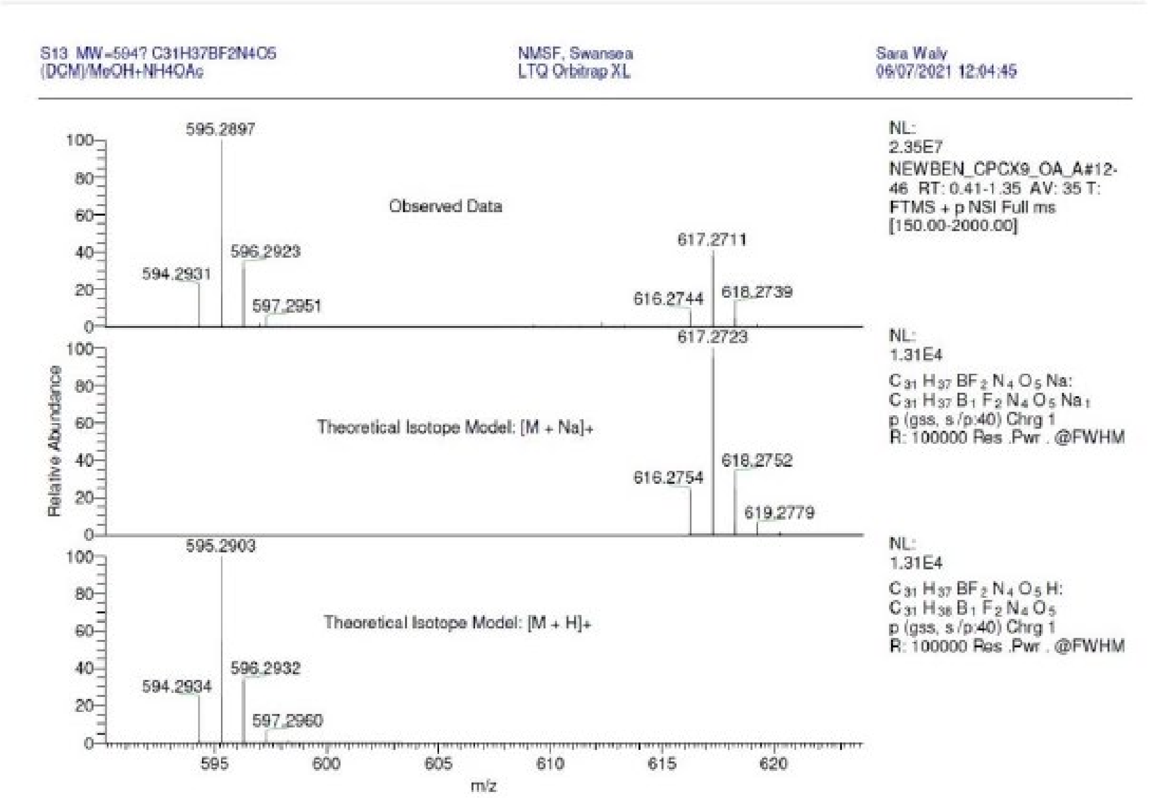

Figure S13. Observed and theoretical mass spectra for **B-P$_1$** showing its $[M+Na]^+$ and $[M+H]^+$ ions.

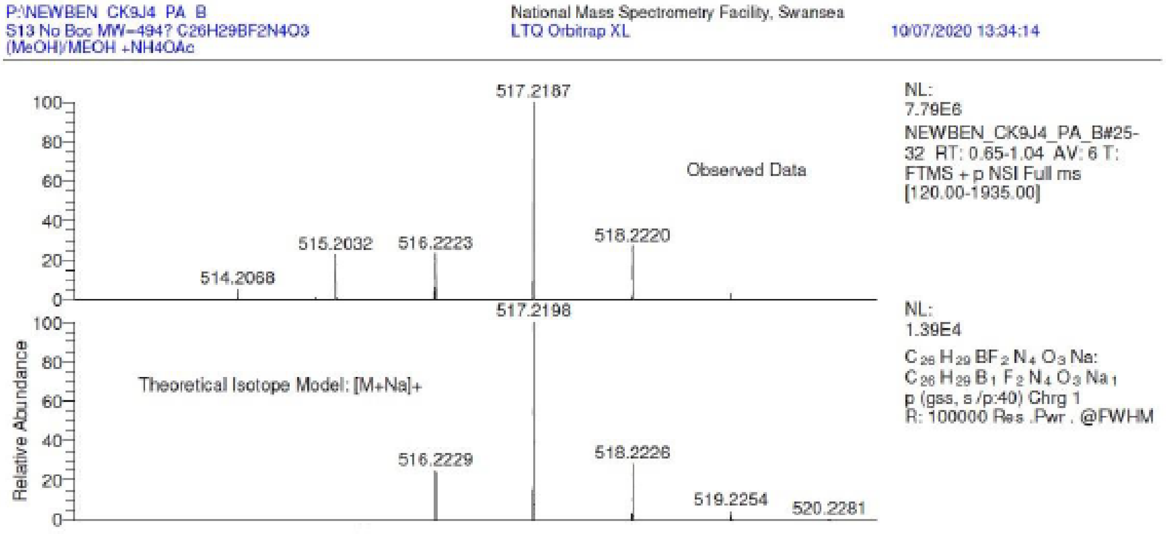

Figure S14. Observed and theoretical mass spectra for **B-P$_1$-NH** showing its $[M+Na]^+$ ion.

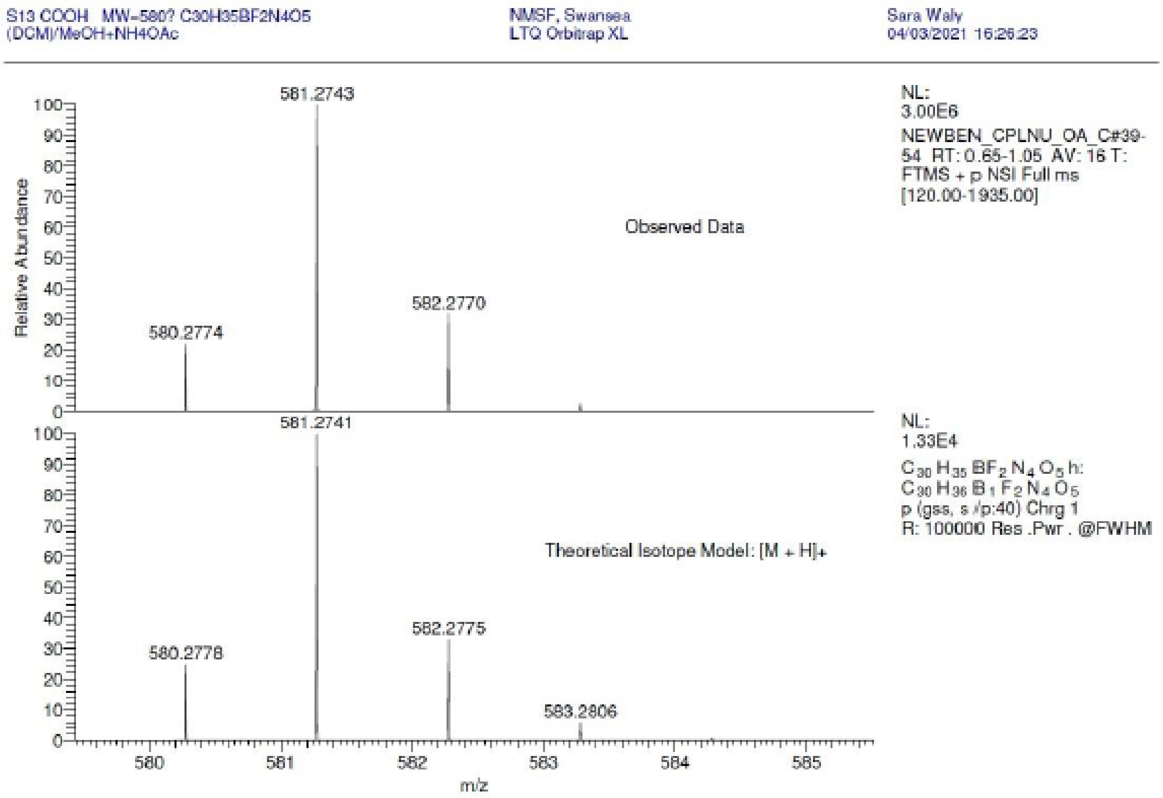

Figure S15. Observed and theoretical mass spectra for **B-P$_1$-COOH** showing its $[M+H]^+$ ion.

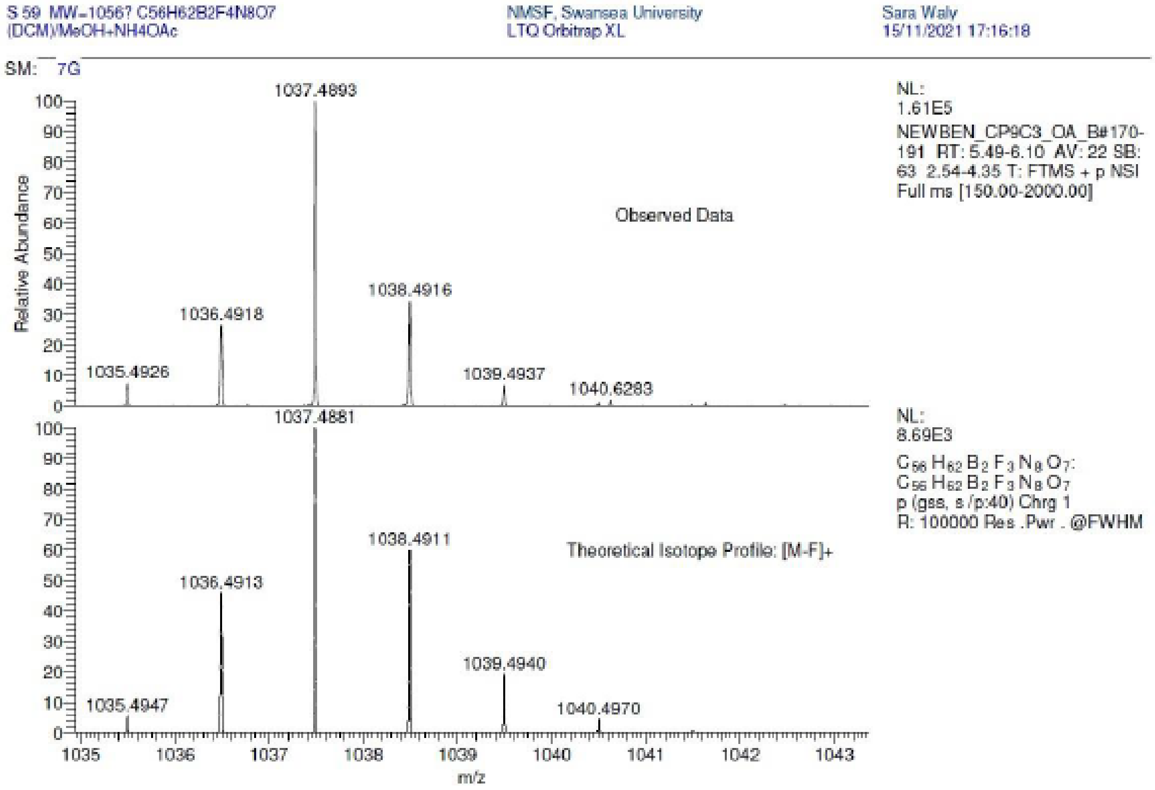

Figure S16. Observed and theoretical mass spectra for **B-P$_1$-B** showing its $[M-F]^+$ ion.

S2. X-ray crystallography and quantum chemical calculations

Table S1: Crystal data and structure refinement for B-P_1.

Identification code	B-P_1
Empirical formula	$C_{64}H_{80}B_2F_4N_8O_{11}$
Formula weight	1234.98
Temperature/K	150.0(2)
Crystal system	orthorhombic
Space group	P212121
a/Å	9.9148(3)
b/Å	16.7335(5)
c/Å	38.6191(9)
α/°	90
β/°	90
γ/°	90
Volume/Å^3	6407.3(3)
Z	4
ρ calcg/cm^3	1.280
μ /mm	0.787
F(000)	2616.0
Crystal size/mm^3	0.22 × 0.12 × 0.05
Radiation	CuKα (λ = 1.54184)
2Θ range for data collection/°	6.99 to 133.628
Index ranges	-11 ≤ h ≤ 9, -19 ≤ k ≤ 13, -45 ≤ l ≤ 40
Reflections collected	27637
Independent reflections	10876 [Rint = 0.0456, Rsigma = 0.0500]
Data/restraints/parameters	10876/69/856
Goodness-of-fit on F2	1.026
Final R indexes [I>=2σ (I)]	R1 = 0.0396, wR2 = 0.0875

Final R indexes [all data] R1 = 0.0525, wR2 = 0.0948

Largest diff. peak/hole / e Å-3 0.20/-0.16

Flack parameter -0.02(8)

Special details of refinement

The ethanol molecule in the asymmetric unit of this structure has been modelled as disordered over two positions. The occupancies of the two sites were refined independently of the atomic displacement parameters. The ethanol geometry was restrained using the SADI card and the displacement parameters of all partially-occupied non-hydrogen atoms were restrained using the SIMU card.

Table S2: Fractional Atomic Coordinates (×104) and Equivalent Isotropic Displacement Parameters (Å2×103). Ueq is defined as 1/3 of of the trace of the orthogonalised UIJ tensor.

Atom	x	y	z	U(eq)
F1	5889(2)	6786.0(12)	5705.0(5)	40.0(5)
F2	3688(2)	6495.6(11)	5606.8(5)	38.2(5)
F3	10842(2)	6525.0(12)	-593.0(5)	38.6(5)
F4	13044(2)	6881.5(12)	-628.1(4)	39.1(5)
O1	3017(2)	6751.4(16)	3094.1(5)	38.6(6)
O2	8027(2)	7773.0(16)	1867.5(6)	39.9(6)
O3	6304(2)	8426.9(14)	1621.9(5)	35.6(5)
O4	6231(3)	6400.8(15)	1541.5(5)	41.4(6)
O5	6349(3)	5406.7(14)	1945.8(5)	37.7(6)
O6	12857(2)	6793.2(17)	1999.5(5)	41.9(6)
O7	7706(2)	7960.2(15)	3111.2(6)	36.2(5)
O8	9576(2)	8354.9(16)	3382.9(6)	41.3(6)
O9	8631(3)	6294.9(14)	3425.9(5)	37.0(6)
O10	8220(2)	5421.3(14)	2983.5(5)	31.7(5)
N1	5315(3)	6111.7(16)	5172.0(6)	26.8(6)
N2	4696(3)	7534.5(15)	5266.3(6)	25.2(6)
N3	5111(3)	7212.9(18)	2988.1(6)	30.1(6)
N4	6019(3)	6655.3(17)	2112.2(6)	28.4(6)
N5	12319(3)	6141.1(16)	-124.9(6)	26.3(6)
N6	11653(3)	7557.4(15)	-210.7(6)	25.8(6)
N7	10642(3)	7061.9(18)	2038.8(6)	29.2(6)
N8	9150(3)	6598.9(17)	2871.6(6)	30.0(6)
C1	5570(3)	5500(2)	4647.2(8)	29.7(7)
C2	5833(4)	4961(2)	4905.8(8)	33.4(8)
C3	5667(3)	5344(2)	5227.4(8)	29.0(7)
C5	4408(3)	8241(2)	5415.5(8)	29.5(7)
C6	4134(4)	8805(2)	5157.4(8)	34.3(8)

C7	4267(3)	8438.8(19)	4836.8(8)	28.7(7)
C8	4872(3)	6988.2(19)	4686.1(7)	25.2(7)
C9	5238(3)	6238.4(19)	4813.7(7)	25.8(7)
C10	4612(3)	7631.4(19)	4906.0(7)	25.9(7)
C11	5706(4)	5315(2)	4267.7(8)	35.0(8)
C12	5827(4)	4992(2)	5577.6(8)	37.5(8)
C13	4431(4)	8365(2)	5799.8(8)	35.4(8)
C14	4110(4)	8846(2)	4492.6(8)	35.1(8)
C15	4696(3)	7082.0(18)	4303.6(7)	24.7(6)
C16	5791(3)	7205(2)	4087.3(8)	30.5(7)
C17	5625(3)	7201(2)	3729.4(8)	29.9(7)
C18	4358(3)	7071.2(19)	3585.3(7)	25.4(7)
C19	3255(3)	6987(2)	3802.2(7)	29.6(7)
C20	3420(3)	6993(2)	4159.1(7)	29.2(7)
C21	4110(3)	6998.3(19)	3201.3(7)	27.0(7)
C22	4975(3)	7149(2)	2613.5(7)	30.0(7)
C23	5651(3)	7833(2)	2418.1(7)	30.6(7)
C24	5738(3)	7497.9(19)	2050.4(7)	27.6(7)
C25	5651(4)	6406(2)	2464.0(7)	31.0(8)
C26	6835(4)	7891(2)	1838.1(7)	29.1(7)
C27	7249(5)	8873(3)	1409.6(10)	50.3(11)
C28	6190(4)	6166(2)	1837.3(8)	31.1(7)
C29	6633(5)	4765(2)	1694.8(8)	43.5(9)
C30	6883(6)	4052(2)	1927.7(10)	64.0(14)
C31	7910(6)	4954(3)	1496.1(13)	72.0(16)
C32	5444(6)	4641(3)	1460.7(13)	77.3(16)
C33	12599(3)	5519.4(19)	397.1(8)	28.8(7)
C34	12859(4)	4986(2)	132.2(9)	33.7(8)
C35	12691(3)	5377(2)	-183.3(8)	30.0(7)
C37	11368(3)	8270(2)	-357.3(8)	30.3(7)
C38	11112(4)	8829(2)	-96.7(8)	32.9(8)
C39	11266(3)	8459.5(19)	222.6(8)	28.1(7)
C40	11877(3)	7002.2(19)	366.7(7)	24.8(7)
C41	12251(3)	6259.2(19)	233.5(7)	24.6(7)
C42	11591(3)	7648.8(19)	149.6(7)	25.3(7)
C43	12722(4)	5323(2)	774.6(8)	36.3(8)
C44	12857(4)	5034(2)	-537.7(9)	38.1(8)
C45	11399(4)	8401(2)	-740.6(8)	35.9(8)
C46	11158(4)	8856(2)	567.6(9)	36.6(8)
C47	11797(3)	7097.9(19)	749.5(7)	25.6(7)
C48	10574(3)	7008.4(19)	922.7(7)	27.9(7)
C49	10539(3)	6994(2)	1282.7(7)	26.7(7)
C50	11713(3)	7066(2)	1472.7(7)	26.2(7)
C51	12926(3)	7194(2)	1299.1(8)	33.4(8)
C52	12955(3)	7225(2)	940.4(8)	32.9(8)
C53	11778(3)	6969(2)	1860.5(7)	28.0(7)
C54	10570(3)	6925(2)	2409.9(7)	29.2(7)

C55	10312(4)	7675(2)	2625.1(8)	33.6(8)
C56	9837(3)	7331(2)	2974.1(7)	28.2(7)
C57	9401(3)	6382(2)	2508.9(7)	29.7(7)
C58	8906(3)	7895(2)	3161.9(7)	28.6(7)
C59	8775(4)	8929(3)	3573.4(10)	50.0(10)
C60	8669(3)	6108(2)	3121.8(8)	30.1(7)
C61	7647(4)	4787(2)	3203.2(8)	35.3(8)
C62	7382(5)	4127(2)	2945.0(10)	51.7(11)
C63	6337(4)	5077(3)	3361.1(10)	50.7(10)
C64	8662(5)	4519(3)	3471.4(11)	59.5(12)
B4	4898(4)	6724(2)	5452.2(8)	28.4(8)
B36	11961(4)	6773(2)	-403.6(8)	27.0(8)
O11A	1675(4)	5308(2)	2942.4(10)	63.4(10)
C65A	2441(11)	4811(5)	2726.3(17)	82(2)
C66A	2275(9)	4836(4)	2346.2(17)	87(2)
O11B	3350(40)	5000(20)	2895(9)	122(10)
C65B	2490(50)	5110(20)	2629(11)	75(9)
C66B	1660(30)	4420(16)	2517(8)	55(7)

*Table S3: Anisotropic Displacement Parameters (Å2×103). The Anisotropic displacement factor exponent takes the form: -2π2[h2a*2U11+2hka*b*U12+…].*

Atom	U11	U22	U33	U23	U13	U12
F1	48.4(12)	44.1(11)	27.5(9)	-1.7(8)	-13.7(8)	1.3(10)
F2	45.5(12)	37.4(11)	31.7(9)	2.1(8)	11.8(9)	-4.8(10)
F3	46.0(12)	39.9(11)	29.8(9)	-0.3(8)	-11.4(8)	-6.4(10)
F4	45.8(12)	42.5(11)	28.9(9)	5.0(8)	13.6(8)	3.2(10)
O1	29.5(13)	59.8(16)	26.5(11)	-0.4(11)	-2.3(9)	-11.6(12)
O2	29.4(14)	51.6(15)	38.6(12)	10.4(11)	2.9(10)	2.5(12)
O3	36.5(13)	40.8(13)	29.4(11)	12.5(10)	0.9(10)	3.3(12)
O4	63.4(17)	42.5(14)	18.2(10)	2.9(10)	3.2(11)	8.4(13)
O5	56.1(16)	34.4(13)	22.8(10)	0.6(10)	2.6(10)	-0.5(13)
O6	27.9(13)	74.9(19)	22.9(10)	4.7(12)	-1.7(9)	1.1(13)
O7	30.6(14)	44.2(14)	33.9(11)	-1.5(11)	-0.4(10)	-3.8(12)
O8	35.6(14)	55.4(15)	32.9(12)	-19.9(12)	-1.4(10)	-3.9(13)
O9	52.6(16)	39.8(13)	18.7(10)	-1.0(9)	2.7(10)	-11.3(12)
O10	41.9(14)	32.9(12)	20.3(10)	-1.5(9)	4.3(9)	-7.7(11)
N1	28.6(15)	29.5(14)	22.2(12)	2.6(11)-	2.8(11)	0.2(12)
N2	28.9(14)	27.7(14)	18.9(11)	-1.4(10)	1.4(10)-	0.5(12)
N3	25.7(14)	47.7(17)	17.1(11)	-1.0(11)	-0.1(10)	-4.5(14)
N4	35.2(16)	33.6(15)	16.5(11)	3.0(11)	1.7(10)	1.1(13)
N5	28.0(14)	28.6(14)	22.4(12)	0.4(11)	0.8(11)	0.3(12)
N6	28.7(14)	28.4(14)	20.3(12)	2.7(11)	-1.0(10)	-1.0(12)
N7	27.2(15)	41.0(17)	19.4(12)	3.0(12)	-0.5(11)	2.2(14)
N8	37.5(16)	34.7(15)	17.8(12)	-2.7(11)	2.4(11)	-8.5(14)
C1	27.0(18)	30.7(18)	31.3(16)	-2.7(13)	0.7(13)	2.7(15)

C2	34(2)	28.0(17)	37.9(17)	0.6(15)	0.3(15)	3.4(15)
C3	25.4(17)	30.0(17)	31.5(15)	5.2(14)	-2.1(13)	-0.5(15)
C5	30.2(18)	31.3(18)	27.0(15)	-5.2(13)	1.4(13)	-4.3(16)
C6	43(2)	29.0(17)	31.0(16)	-4.4(14)	-0.8(15)	2.0(16)
C7	32.2(18)	27.4(17)	26.7(15)	0.2(13)	-0.6(13)	-2.5(15)
C8	23.5(16)	30.2(17)	21.8(14)	-0.3(13)	0.6(12)	-2.0(14)
C9	27.9(17)	30.0(16)	19.7(13)	0.3(13)	-0.1(12)	-0.3(14)
C10	28.7(17)	30.2(17)	18.9(13)	-1.3(12)	-0.7(12)	-0.7(15)
C11	40(2) 3	4.3(18)	30.3(16)	-7.4(15)	3.9(15)	3.9(17)
C12	42(2)	37.4(19)	33.2(17)	10.1(15)	-5.3(15)	1.8(17)
C13	39(2)	42(2) 2	5.1(15)	-8.9(15)	4.0(14)	-2.0(18)
C14	50(2)	27.0(17)	28.4(16)	1.6(14)	-4.7(15)	0.3(17)
C15	29.5(17)	25.2(15)	19.3(13)	0.1(12)	1.4(12)	1.9(14)
C16	22.8(16)	42.6(19)	26.1(15)	0.9(14)	-1.5(12)	-3.4(16)
C17	26.0(17)	42.2(19)	21.6(14)	1.5(13)	3.2(12)	-4.2(16)
C18	29.7(17)	26.7(16)	19.7(14)	1.2(12)	1.0(12)	2.2(15)
C19	23.7(16)	42.5(19)	22.7(14)	1.3(14)	-1.4(12)	-1.3(16)
C20	25.6(18)	39.6(19)	22.6(14)	1.4(13)	5.3(12)	1.8(16)
C21	28.9(18)	29.7(17)	22.5(14)	-0.4(13)	-0.6(12)	1.5(15)
C22	25.7(17)	47(2)	17.0(13)	-0.7(14)	-0.7(12)	-2.5(16)
C23	32.7(18)	37.7(18)	21.4(14)	-0.2(13)	-0.3(13)	2.7(16)
C24	28.2(18)	34.9(18)	19.9(14)	4.0(13)	-0.7(12)	0.8(15)
C25	36.7(19)	37.9(19)	18.3(14)	3.3(13)	4.2(13)	-3.9(16)
C26	32.5(19)	33.5(17)	21.3(14)	-0.6(13)	0.0(13)	4.2(16)
C27	60(3)	49(2)	42(2)	19.5(18)	7.4(18)	-7(2)
C28	34.3(19)	35.1(19)	24.0(16)	2.1(14)	0.3(13)	2.3(16)
C29	68(3) 3	8(2)	24.2(15)	-6.2(15)	2.8(17)	2(2)
C30	122(4)	34(2)	36(2)	1.7(17)	10(2)	3(3)
C31	99(4)	54(3)	63(3)	9(2)	40(3)	27(3)
C32	103(5)	69(3)	60(3)	-20(3)	-29(3)	1(3)
C33	25.7(18)	31.5(18)	29.1(15)	5.3(13)	0.5(13)	0.6(15)
C34	32(2)	28.0(17)	40.9(18)	3.0(14)	2.9(15)	4.2(15)
C35	28.1(18)	28.6(17)	33.4(16)	-2.1(14)	4.1(13)	-1.6(15)
C37	28.0(18)	33.1(18)	29.8(16)	6.5(14)-	4.1(13)	-1.9(16)
C38	38(2)	26.3(17)	34.2(17)	2.3(14)	-2.6(15)	-0.2(16)
C39	29.6(18)	25.8(16)	28.9(15)	1.5(13)	2.1(13)	-1.2(14)
C40	21.4(16)	30.7(18)	22.4(14)	1.4(13)	0.3(11)	-2.1(14)
C41	25.9(17)	28.7(16)	19.0(13)	3.3(12)	1.4(12)	-1.4(14)
C42	23.1(16)	30.3(17)	22.4(14)	-0.7(13)	1.3(12)	-1.7(14)
C43	41(2)	32.9(18)	35.3(17)	12.0(15)	-3.6(15)	0.4(17)
C44	44(2)	36.3(19)	34.5(17)	-7.3(15)	1.0(15)	0.9(17)
C45	38(2)	39.7(19)	30.2(16)	11.4(15)	-5.5(14)	-2.6(17)
C46	47(2)	28.3(18)	34.2(17)	-3.8(14)	3.0(16)	3.9(17)
C47	29.1(17)	27.1(16)	20.5(14)	1.8(12)	-0.2(12)	0.9(15)
C48	25.2(17)	33.9(18)	24.5(14)	1.6(13)	-2.2(12)	-0.7(15)
C49	22.2(16)	35.9(18)	22.0(13)	0.1(13)	1.5(12)	-1.1(15)
C50	26.3(17)	32.5(17)	19.9(13)	1.1(13)	1.8(12)	0.0(15)

C51	24.3(17)	54(2)	22.2(14)	2.0(15)	-2.9(12)	-4.8(17)
C52	23.2(17)	51(2)	24.3(15)	5.2(15)	3.6(13)	-5.8(16)
C53	28.7(18)	34.7(18)	20.5(14)	-0.1(13)	-0.9(13)	-4.9(15)
C54	30.3(17)	39.6(18)	17.7(13)	2.3(13)	1.6(12)	1.5(15)
C55	38.0(19)	38.3(19)	24.3(14)	1.0(14)	3.7(14)	-9.8(16)
C56	30.6(18)	34.7(18)	19.4(13)	0.0(13)	2.1(12)	-5.0(15)
C57	34.3(18)	38.4(19)	16.4(13)	-2.4(13)	3.6(13)	-4.9(16)
C58	34(2)	36.6(19)	15.6(13)	2.1(13)	1.8(12)	-8.7(16)
C59	51(3)	57(3)	42(2) -	22.9(19)	1.7(18)	6(2)
C60	34.4(19)	33.3(18)	22.5(15)	0.6(13)	-0.3(13)	-3.6(16)
C61	47(2)	31.1(18)	27.6(15)	3.6(14)	4.4(15)	-8.5(17)
C62	72(3)	40(2)	43(2)	-8.6(17)	9(2)	-14(2)
C63	51(3)	55(2)	47(2)	-1.5(19)	20.0(19)	-12(2)
C64	78(3)	49(2)	51(2)	15(2)	-14(2)	-6(2)
B4	33(2)	34(2)	18.4(14)	2.3(14)	0.8(14)-	1.3(18)
B36	32(2)	31(2)	18.4(15)	1.9(14)	-0.8(14)	-0.5(17)
O11A	59(2)	61(2)	70(2)	4(2)	1.2(19)	-4(2)
C65A	116(6)	77(5)	52(4)	8(4)	6(4)	18(6)
C66A	115(6)	70(4)	78(4)	-18(4) -	10(4)	17(4)
O11B	110(20)	160(20)	98(19)	15(19) -	21(17)	10(20)
C65B	75(16)	77(18)	73(17)	17(16)	8(16)	14(16)
C66B	56(16)	42(14)	67(16)	3(13)	-13(14)	-9(13)

Table S4: Bond Lengths.

Atom	Atom	Length/Å	Atom	Atom	Length/Å
F1	B4	1.389(4)	C7	C14	1.502(4)
F2	B4	1.394(4)	C8	C9	1.396(5)
F3	B36	1.393(4)	C8	C10	1.395(4)
F4	B36	1.392(4)	C8	C15	1.496(4)
O1	C21	1.231(4)	C15	C16	1.385(4)
O2	C26	1.204(4)	C15	C20	1.391(4)
O3	C26	1.334(4)	C16	C17	1.392(4)
O3	C27	1.451(4)	C17	C18	1.391(5)
O4	C28	1.209(4)	C18	C19	1.384(4)
O5	C28	1.347(4)	C18	C21	1.508(4)
O5	C29	1.474(4)	C19	C20	1.388(4)
O6	C53	1.232(4)	C22	C23	1.526(5)
O7	C58	1.211(4)	C22	C25	1.525(5)
O8	C58	1.327(4)	C23	C24	1.529(4)
O8	C59	1.447(4)	C24	C26	1.513(5)
O9	C60	1.216(4)	C29	C30	1.514(5)
O10	C60	1.343(4)	C29	C31	1.514(7)
O10	C61	1.473(4)	C29	C32	1.500(6)
N1	C3	1.349(4)	C33	C34	1.382(5)
N1	C9	1.402(4)	C33	C41	1.432(4)

N1	B4	1.546(5)	C33	C43	1.499(4)
N2	C5	1.346(4)	C34	C35	1.392(5)
N2	C10	1.403(4)	C35	C44	1.493(4)
N2	B4	1.548(5)	C37	C38	1.397(5)
N3	C21	1.339(4)	C37	C45	1.496(4)
N3	C22	1.457(4)	C38	C39	1.388(5)
N4	C24	1.457(4)	C39	C42	1.423(5)
N4	C25	1.467(4)	C39	C46	1.492(4)
N4	C28	1.352(4)	C40	C41	1.396(5)
N5	C35	1.350(4)	C40	C42	1.398(4)
N5	C41	1.400(4)	C40	C47	1.489(4)
N5	B36	1.550(4)	C47	C48	1.393(4)
N6	C37	1.350(4)	C47	C52	1.381(4)
N6	C42	1.401(4)	C48	C49	1.391(4)
N6	B36	1.540(5)	C49	C50	1.381(4)
N7	C53	1.329(4)	C50	C51	1.394(4)
N7	C54	1.453(4)	C50	C53	1.508(4)
N8	C56	1.456(4)	C51	C52	1.387(4)
N8	C57	1.468(4)	C54	C55	1.527(5)
N8	C60	1.355(4)	C54	C57	1.522(5)
C1	C2	1.371(5)	C55	C56	1.540(4)
C1	C9	1.432(5)	C56	C58	1.507(5)
C1	C11	1.504(4)	C61	C62	1.511(5)
C2	C3	1.407(5)	C61	C63	1.515(5)
C3	C12	1.483(4)	C61	C64	1.513(6)
C5	C6	1.399(5)	O11A	C65A	1.402(9)
C5	C13	1.499(4)	C65A	C66A	1.478(9)
C6	C7	1.388(5)	O11B	C65B	1.35(3)
C7	C10	1.419(5)	C65B	C66B	1.49(2)

Table S5: Bond Angles.

Atom	Atom	Atom	Angle/°	Atom	Atom	Atom	Angle/°
C26	O3	C27	116.4(3)	O5	C29	C31	109.9(3)
C28	O5	C29	120.3(2)	O5	C29	C32	110.3(4)
C58	O8	C59	115.9(3)	C31	C29	C30	109.2(4)
C60	O10	C61	121.0(2)	C32	C29	C30	112.2(4)
C3	N1	C9	108.3(3)	C32	C29	C31	112.4(4)
C3	N1	B4	126.1(3)	C34	C33	C41	106.0(3)
C9	N1	B4	125.2(3)	C34	C33	C43	124.3(3)
C5	N2	C10	108.1(3)	C41	C33	C43	129.6(3)
C5	N2	B4	126.8(2)	C33	C34	C35	108.8(3)
C10	N2	B4	124.7(3)	N5	C35	C34	109.3(3)
C21	N3	C22	121.5(3)	N5	C35	C44	123.2(3)
C24	N4	C25	112.3(2)	C34	C35	C44	127.5(3)
C28	N4	C24	118.8(2)	N6	C37	C38	109.1(3)

C28	N4	C25	125.9(3)	N6	C37	C45	122.7(3)
C35	N5	C41	108.2(3)	C38	C37	C45	128.2(3)
C35	N5	B36	126.4(3)	C39	C38	C37	108.8(3)
C41	N5	B36	125.4(3)	C38	C39	C42	105.9(3)
C37	N6	C42	108.1(3)	C38	C39	C46	126.0(3)
C37	N6	B36	126.2(2)	C42	C39	C46	128.1(3)
C42	N6	B36	125.6(2)	C41	C40	C42	121.5(3)
C53	N7	C54	122.3(3)	C41	C40	C47	118.4(3)
C56	N8	C57	112.9(2)	C42	C40	C47	120.1(3)
C60	N8	C56	118.7(2)	N5	C41	C33	107.6(3)
C60	N8	C57	126.1(3)	C40	C41	N5	120.2(3)
C2	C1	C9	106.6(3)	C40	C41	C33	132.2(3)
C2	C1	C11	123.9(3)	N6	C42	C39	108.1(3)
C9	C1	C11	129.5(3)	C40	C42	N6	120.2(3)
C1	C2	C3	108.7(3)	C40	C42	C39	131.7(3)
N1	C3	C2	108.9(3)	C48	C47	C40	120.7(3)
N1	C3	C12	123.4(3)	C52	C47	C40	120.2(3)
C2	C3	C12	127.7(3)	C52	C47	C48	119.0(3)
N2	C5	C6	109.2(3)	C49	C48	C47	120.2(3)
N2	C5	C13	122.8(3)	C50	C49	C48	120.5(3)
C6	C5	C13	128.0(3)	C49	C50	C51	119.0(3)
C7	C6	C5	108.6(3)	C49	C50	C53	123.7(3)
C6	C7	C10	106.0(3)	C51	C50	C53	117.2(3)
C6	C7	C14	125.4(3)	C52	C51	C50	120.3(3)
C10	C7	C14	128.6(3)	C47	C52	C51	120.7(3)
C9	C8	C15	118.2(3)	O6	C53	N7	122.5(3)
C10	C8	C9	121.8(3)	O6	C53	C50	119.7(3)
C10	C8	C15	119.9(3)	N7	C53	C50	117.7(3)
N1	C9	C1	107.5(3)	N7	C54	C55	114.6(3)
C8	C9	N1	119.9(3)	N7	C54	C57	112.3(3)
C8	C9	C1	132.6(3)	C57	C54	C55	103.1(3)
N2	C10	C7	108.1(3)	C54	C55	C56	102.7(3)
C8	C10	N2	120.2(3)	N8	C56	C55	102.7(2)
C8	C10	C7	131.6(3)	N8	C56	C58	111.9(3)
C16	C15	C8	121.3(3)	C58	C56	C55	112.0(3)
C16	C15	C20	119.1(3)	N8	C57	C54	102.7(2)
C20	C15	C8	119.4(3)	O7	C58	O8	122.9(3)
C15	C16	C17	120.4(3)	O7	C58	C56	125.5(3)
C18	C17	C16	120.3(3)	O8	C58	C56	111.5(3)
C17	C18	C21	123.6(3)	O9	C60	O10	126.4(3)
C19	C18	C17	119.1(3)	O9	C60	N8	122.9(3)
C19	C18	C21	117.3(3)	O10	C60	N8	110.6(3)
C18	C19	C20	120.5(3)	O10	C61	C62	102.3(3)
C19	C20	C15	120.4(3)	O10	C61	C63	109.4(3)
O1	C21	N3	122.4(3)	O10	C61	C64	110.6(3)
O1	C21	C18	120.1(3)	C62	C61	C63	110.5(3)
N3	C21	C18	117.5(3)	C62	C61	C64	110.6(3)

N3	C22	C23	113.3(3)	C64	C61	C63	112.9(3)
N3	C22	C25	113.3(3)	F1	B4	F2	109.2(2)
C25	C22	C23	103.4(2)	F1	B4	N1	110.6(3)
C22	C23	C24	102.1(3)	F1	B4	N2	110.6(3)
N4	C24	C23	102.3(2)	F2	B4	N1	110.4(3)
N4	C24	C26	111.9(3)	F2	B4	N2	109.1(3)
C26	C24	C23	112.6(3)	N1	B4	N2	106.9(2)
N4	C25	C22	103.2(3)	F3	B36	N5	110.1(3)
O2	C26	O3	123.9(3)	F3	B36	N6	110.5(3)
O2	C26	C24	125.7(3)	F4	B36	F3	109.1(2)
O3	C26	C24	110.4(3)	F4	B36	N5	110.2(3)
O4	C28	O5	126.7(3)	F4	B36	N6	110.1(3)
O4	C28	N4	123.3(3)	N6	B36	N5	106.9(2)
O5	C28	N4	110.0(3)	O11A	C65A	C66A	120.9(7)
O5	C29	C30	102.4(3)	O11B	C65B	C66B	118(3)

Table S6: Hydrogen Bonds.

D	H	A	d(D-H)/Å	d(H-A)/Å	d(D-A)/Å	D-H-A/°
N3	H3	O7	0.83(4)	2.11(4)	2.900(4)	159(4)
N7	H7	O2	0.81(4)	2.16(4)	2.928(4)	159(4)
O11A	H11D	O1	0.82	2.02	2.819(5)	162.9
O11B	H11E	O1	0.82	2.32	3.05(4)	149.6

Table S7: Hydrogen Atom Coordinates (Å×104) and Isotropic Displacement Parameters ($Å^2$×10^3).

Atom	x	y	z	U(eq)
H3	5830(40)	7380(20)	3076(10)	36
H7	10010(40)	7250(20)	1940(10)	35
H2	6080	4429	4874	40
H6	3902	9337	5194	41
H11A	6537	5532	4182	53
H11B	4964	5548	4144	53
H11C	5702	4746	4235	53
H12A	6647	5185	5680	56
H12B	5864	4420	5560	56
H12C	5075	5143	5720	56
H13A	5330	8499	5872	53
H13B	4150	7883	5914	53
H13C	3829	8792	5860	53
H14A	4922	8781	4360	53
H14B	3941	9405	4528	53
H14C	3367	8613	4369	53
H16	6641	7291	4182	37

H17	6366	7284	3586	36
H19	2398	6926	3708	36
H20	2672	6937	4302	35
H22	4015	7138	2554	36
H23A	6540	7947	2511	37
H23B	5108	8314	2426	37
H24	4866	7557	1934	33
H25A	6444	6260	2597	37
H25B	5033	5957	2459	37
H27A	6760	9196	1247	75
H27B	7821	8507	1287	75
H27C	7790	9211	1555	75
H30A	7619	4167	2081	96
H30B	7101	3594	1789	96
H30C	6086	3943	2061	96
H31A	7765	5418	1354	108
H31B	8143	4508	1352	108
H31C	8630	5056	1656	108
H32A	4653	4543	1598	116
H32B	5609	4190	1313	116
H32C	5309	5110	1322	116
H34	13105	4454	160	40
H38	10877	9361	-131	39
H43A	13525	5564	866	55
H43B	12770	4754	803	55
H43C	11950	5526	896	55
H44A	12001	5030	-654	57
H44B	13194	4498	-520	57
H44C	13483	5354	-667	57
H45A	12316	8465	-815	54
H45B	10894	8873	-797	54
H45C	11006	7948	-855	54
H46A	10424	8625	695	55
H46B	10998	9417	536	55
H46C	11983	8781	694	55
H48	9778	6958	797	33
H49	9718	6935	1396	32
H51	13719	7260	1424	40
H52	13763	7332	827	39
H54	11415	6679	2487	35
H55A	9621	8007	2521	40
H55B	11130	7987	2653	40
H56	10617	7203	3120	34
H57A	8615	6485	2366	36
H57B	9649	5823	2488	36
H59A	8429	9324	3417	75
H59B	9327	9184	3745	75

H59C	8038	8660	3685	75
H62A	6767	4315	2771	78
H62B	6994	3676	3062	78
H62C	8215	3971	2838	78
H63A	6521	5502	3521	76
H63B	5907	4643	3481	76
H63C	5752	5268	3181	76
H64A	9511	4414	3361	89
H64B	8344	4041	3582	89
H64C	8776	4931	3642	89
H11D	2153	5670	3017	95
H65A	2272	4265	2799	98
H65B	3383	4921	2775	98
H66A	2778	4408	2243	131
H66B	2602	5338	2260	131
H66C	1338	4780	2289	131
H11E	3557	5431	2980	182
H65C	3004	5293	2431	90
H65D	1878	5542	2692	90
H66D	884	4609	2391	82
H66E	1361	4126	2716	82
H66F	2184	4078	2371	82

Table S8: Atomic Occupancy for B-P$_1$.

Atom	Occupancy	Atom	Occupancy	Atom	Occupancy
O11A	0.8473	H11D	0.8473	C65A	0.8473
H65A	0.8473	H65B	0.8473	C66A	0.8473
H66A	0.8473	H66B	0.8473	H66C	0.8473
O11B	0.1527	H11E	0.1527	C65B	0.1527
H65C	0.1527	H65D	0.1527	C66B	0.1527
H66D	0.1527	H66E	0.1527	H66F	0.1527

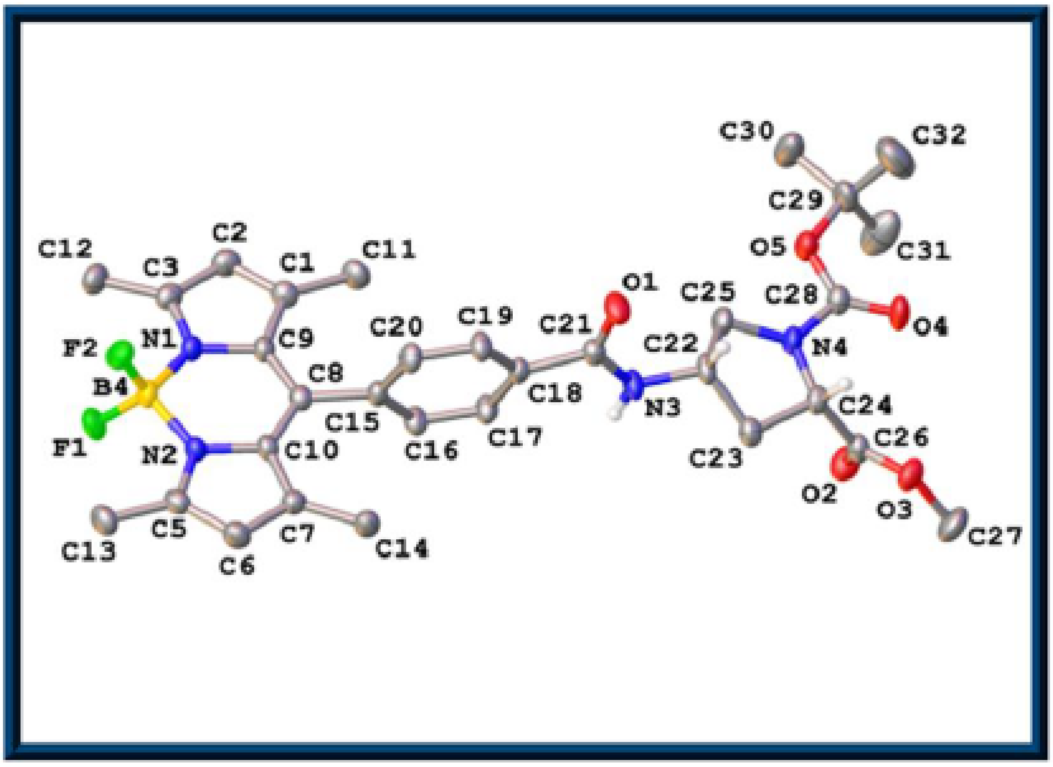

Figure S17. Structure derived for B-P_1 and the atom labelling scheme.

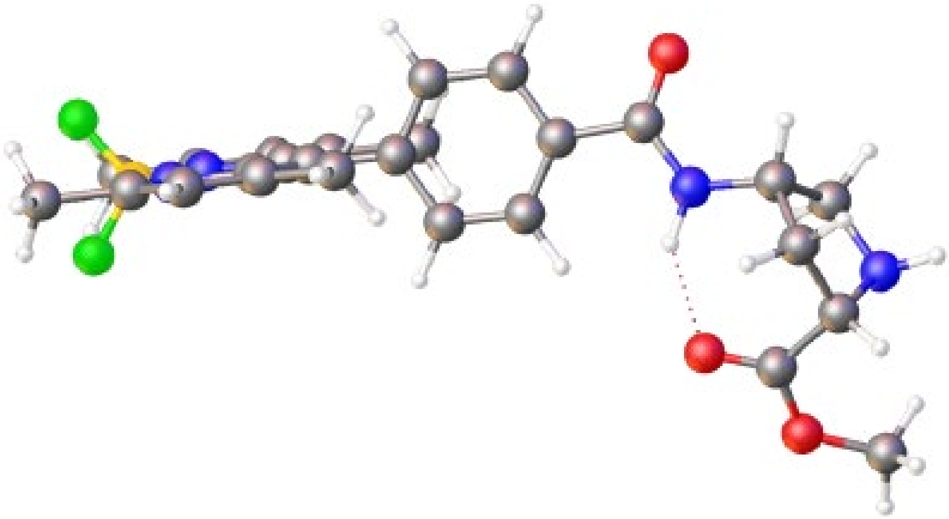

Figure S18. Structure derived for B-P_1 by quantum chemical calculation with the solvent embedded in a reservoir of $CHCl_3$ molecules. Hydrogen bonding between N-H and C=O is highlighted.

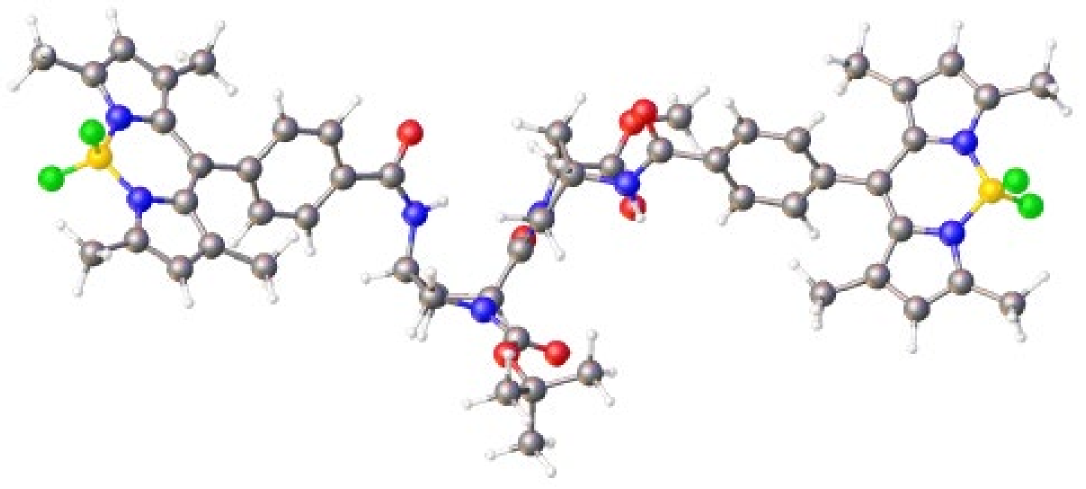

Figure S19. Structure computed for B-P_2-B in a reservoir of $CHCl_3$ molecules.

Table S9. Cartesian coordinates for the energy-minimized structure derived for B-P_1.

C2	C	0.18277	0.09076	-0.05683
C3	C	0.16556	0.21586	-0.12908
C4	C	0.23126	0.26049	-0.20652
H5	H	0.23809	0.35151	-0.27297
C6	C	0.28806	0.16511	-0.18289
N7	N	0.25943	0.06485	-0.09445
C8	C	0.13980	0.00079	0.03493
C9	C	0.17095	-0.11666	0.09204
C10	C	0.14048	-0.22405	0.18669
H11	H	0.19490	-0.40671	0.27147
C12	C	0.19849	-0.31455	0.20639
C13	C	0.26359	-0.26450	0.12563
N14	N	0.24733	-0.14760	0.05812
C15	C	0.05855	0.03102	0.07314
C16	C	-0.09245	0.08684	0.14362
C17	C	0.03939	0.09761	0.22174
C18	C	0.00225	-0.00778	-0.03982
C19	C	-0.07314	0.01999	-0.00482
C20	C	-0.03587	0.12500	0.25640
H21	H	0.08310	0.12734	0.30902
H22	H	0.01730	-0.05955	-0.15440
H23	H	-0.11548	-0.01176	-0.09419
H24	H	-0.05223	0.17590	0.37040
B25	B	0.30418	-0.06025	-0.04195

F26	F	0.36651	-0.02690	0.06116
F27	F	0.33128	-0.12684	-0.18689
C28	C	0.09173	0.29051	-0.12648
H29	H	0.04686	0.23494	-0.18776
H30	H	0.09856	0.38307	-0.19372
H31	H	0.07381	0.31142	0.00306
C32	C	0.06137	-0.24198	0.25549
H33	H	0.01980	-0.24191	0.15353
H34	H	0.04644	-0.16377	0.34341
H35	H	0.05771	-0.33517	0.32250
C36	C	0.36881	0.16682	-0.24214
H37	H	0.37920	0.08196	-0.32293
H38	H	0.40622	0.15864	-0.13272
H39	H	0.38107	0.25642	-0.31167
C40	C	0.34080	-0.32490	0.11102
H41	H	0.35537	-0.33613	-0.02273
H42	H	0.34256	-0.41967	0.17409
H43	H	0.38242	-0.25934	0.16739
C44	C	-0.17233	0.12142	0.19394
O45	O	-0.18549	0.17521	0.33117
N46	N	-0.22715	0.09081	0.08119
H47	H	-0.21676	0.04921	-0.03204
C48	C	-0.30699	0.11835	0.11368
C49	C	-0.38781	0.04899	-0.12384
C50	C	-0.35190	-0.01067	0.15948
H51	H	-0.30940	0.18889	0.21638
H52	H	-0.43628	0.07473	-0.20169
H53	H	-0.31328	-0.09322	0.15520
H54	H	-0.37731	-0.00590	0.28509
C55	C	-0.33446	-0.03981	-0.22470
O56	O	-0.26638	-0.02317	-0.22920
C57	C	-0.34650	0.16990	-0.05066
H58	H	-0.38865	0.24324	-0.01673
H59	H	-0.30614	0.21304	-0.13798
N60	N	-0.41226	-0.02788	0.02774
H61	H	-0.46374	0.00072	0.06928
O62	O	-0.36448	-0.14139	-0.31026
C63	C	-0.44335	-0.18226	-0.30037
H64	H	-0.45904	-0.19909	-0.16929
H65	H	-0.44621	-0.27186	-0.37388
H66	H	-0.48015	-0.10890	-0.35714

Table S10. Cartesian coordinates for the energy-minimized structure derived for B-P_2-B.

C2	C	-0.15646	0.05473	0.04735
C3	C	-0.14277	-0.01013	0.04142

C4	C	-0.09547	-0.00965	-0.04053
N5	N	-0.06682	0.04717	-0.03529
C6	C	-0.09488	0.08409	0.03536
N7	N	-0.11716	-0.03132	0.13114
C8	C	-0.06185	0.09007	0.13121
O9	O	-0.08851	0.11845	0.19149
C10	C	-0.10631	-0.08653	0.16304
O11	O	-0.07916	-0.09019	0.23877
C12	C	-0.12637	-0.14373	0.12076
C13	C	-0.10188	-0.19486	0.16077
C14	C	-0.12050	-0.24976	0.13131
C15	C	-0.16541	-0.25588	0.06201
C16	C	-0.19035	-0.20589	0.02258
C17	C	-0.17083	-0.15096	0.04993
C18	C	-0.18782	-0.31385	0.03166
C19	C	-0.21195	-0.35709	0.10413
N20	N	-0.23142	-0.40982	0.07604
B21	B	-0.23332	-0.43060	-0.02715
N22	N	-0.20797	-0.38300	-0.09182
C23	C	-0.18714	-0.33054	-0.06276
C24	C	-0.22049	-0.35378	0.20386
C25	C	-0.24280	-0.40663	0.23313
C26	C	-0.24945	-0.43984	0.15321
C27	C	-0.20128	-0.38662	-0.18729
C28	C	-0.17458	-0.33758	-0.22130
C29	C	-0.16469	-0.30162	-0.14452
C30	C	-0.21904	-0.43652	-0.24952
C31	C	-0.13292	-0.24484	-0.15801
C32	C	-0.21172	-0.30645	0.27525
C33	C	-0.27393	-0.50024	0.15206
F34	F	-0.29544	-0.44332	-0.05393
F35	F	-0.19640	-0.48226	-0.03668
C36	C	-0.02733	0.06854	-0.10164
O37	O	-0.01139	0.11892	-0.09643
O38	O	-0.00633	0.03448	-0.17381
C39	C	0.02720	0.05219	-0.25283
C40	C	0.08756	0.08081	-0.22362
C41	C	0.04170	-0.00155	-0.31247
C42	C	-0.01021	0.09424	-0.31431
C43	C	0.05833	0.01337	0.25593
C44	C	0.08621	0.01643	0.15723
C45	C	0.03032	0.02930	0.09633
N46	N	-0.00809	0.06442	0.15638
C47	C	0.01328	0.06364	0.25451
N48	N	0.13071	0.06302	0.15288
C49	C	0.04066	0.12026	0.28497
O50	O	0.04211	0.16142	0.23055

C51	C	0.18678	0.05636	0.19196
O52	O	0.19934	0.01081	0.23141
C53	C	0.23517	0.10125	0.18406
C54	C	0.22392	0.15901	0.15762
C55	C	0.27172	0.19879	0.15081
C56	C	0.33154	0.18123	0.16838
C57	C	0.34238	0.12401	0.19421
C58	C	0.29517	0.08482	0.20295
C59	C	0.38409	0.22094	0.16043
C60	C	0.42330	0.23123	0.24713
N61	N	0.47337	0.26445	0.24238
B62	B	0.49456	0.29567	0.15245
N63	N	0.44965	0.28308	0.07125
C64	C	0.40013	0.24815	0.07701
C65	C	0.41626	0.21318	0.34227
C66	C	0.46487	0.23496	0.39268
C67	C	0.49925	0.26589	0.32932
C68	C	0.45331	0.30370	-0.01860
C69	C	0.40699	0.28205	-0.07247
C70	C	0.37361	0.24593	-0.01467
C71	C	0.50088	0.34367	-0.05712
C72	C	0.32165	0.21273	-0.05635
C73	C	0.36719	0.17882	0.38939
C74	C	0.55650	0.29715	0.35382
F75	F	0.55456	0.27536	0.12605
F76	F	0.49632	0.35745	0.17056
O77	O	0.06253	0.12443	0.37506
C78	C	0.08833	0.17278	0.41537
H79	H	-0.18380	0.06881	0.10942
H80	H	-0.18321	0.06534	-0.01775
H81	H	-0.18601	-0.03125	0.02693
H82	H	-0.11960	-0.01174	-0.10994
H83	H	-0.06321	-0.04626	-0.03633
H84	H	-0.10310	0.12828	0.00726
H85	H	-0.10559	-0.00045	0.18018
H86	H	-0.06813	-0.19284	0.21651
H87	H	-0.10062	-0.28752	0.16414
H88	H	-0.22631	-0.20946	-0.02937
H89	H	-0.19365	-0.11634	0.01598
H90	H	-0.25437	-0.41907	0.30519
H91	H	-0.16297	-0.32907	-0.29481
H92	H	-0.23978	-0.47227	-0.20879
H93	H	-0.17851	-0.45335	-0.28674
H94	H	-0.25238	-0.42158	-0.30319
H95	H	-0.09271	-0.24244	-0.11094
H96	H	-0.16306	-0.20757	-0.14793
H97	H	-0.11688	-0.24069	-0.23264

H98	H	-0.20133	-0.26429	0.24434
H99	H	-0.17630	-0.31920	0.32721
H100	H	-0.25478	-0.30026	0.31459
H101	H	-0.28555	-0.51473	0.22515
H102	H	-0.23964	-0.53012	0.12230
H103	H	-0.31537	-0.50203	0.10766
H104	H	0.11485	0.05103	-0.17915
H105	H	0.08083	0.12138	-0.18340
H106	H	0.11435	0.09230	-0.28786
H107	H	0.06895	-0.03257	-0.27018
H108	H	0.06786	0.01025	-0.37724
H109	H	-0.00050	-0.02341	-0.33517
H110	H	-0.02133	0.13419	-0.27458
H111	H	-0.05322	0.07364	-0.33670
H112	H	0.01518	0.10697	-0.37924
H113	H	0.08969	0.01195	0.31760
H114	H	0.03131	-0.02720	0.25735
H115	H	0.10839	-0.02390	0.13243
H116	H	0.00587	-0.01123	0.07980
H117	H	0.04554	0.05113	0.03074
H118	H	-0.02377	0.05283	0.30497
H119	H	0.12077	0.09964	0.11457
H120	H	0.17874	0.17414	0.14291
H121	H	0.26223	0.24299	0.13091
H122	H	0.38815	0.10944	0.20757
H123	H	0.30610	0.04100	0.22312
H124	H	0.47386	0.22927	0.46783
H125	H	0.39922	0.29054	-0.14728
H126	H	0.53481	0.35588	-0.00279
H127	H	0.47919	0.38365	-0.08384
H128	H	0.52492	0.32227	-0.11682
H129	H	0.28573	0.24310	-0.07957
H130	H	0.30249	0.17943	-0.01028
H131	H	0.33814	0.18890	-0.11977
H132	H	0.32278	0.19972	0.37684
H133	H	0.37492	0.17698	0.46745
H134	H	0.36683	0.13366	0.36516
H135	H	0.56775	0.29137	0.43026
H136	H	0.55135	0.34385	0.33904
H137	H	0.59427	0.27965	0.31057
H138	H	0.13482	0.16208	0.43902
H139	H	0.06131	0.18575	0.47841
H140	H	0.09050	0.20970	0.36520

S3. Spectroscopy in liquid solution

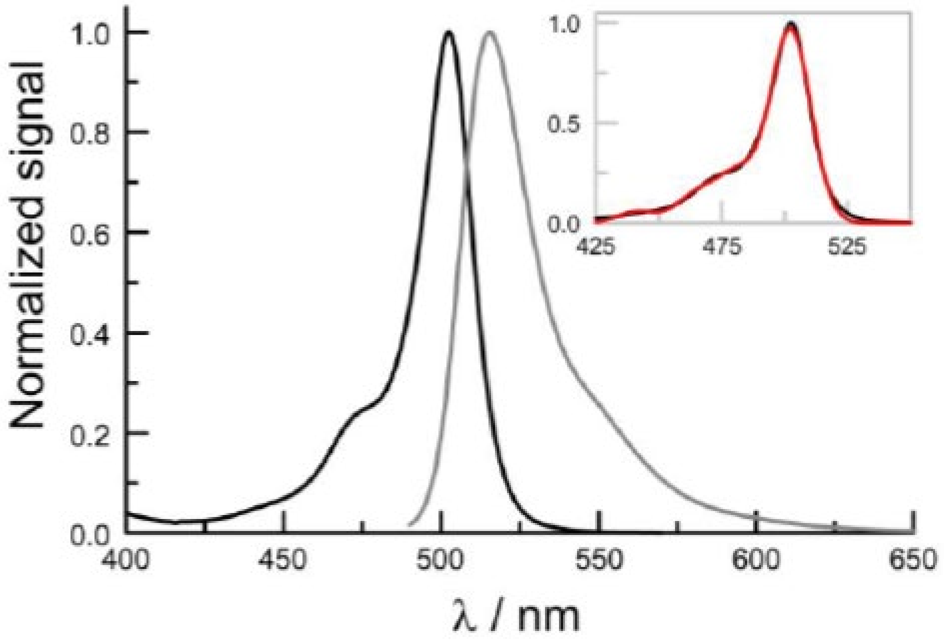

Figure S20. Normalized absorption (black curve) and fluorescence (grey curve) recorded for B-P_1 in CH_2Cl_2 solution. The insert compares absorption (black curve) and excitation (red curve) spectra recorded in dilute solution.

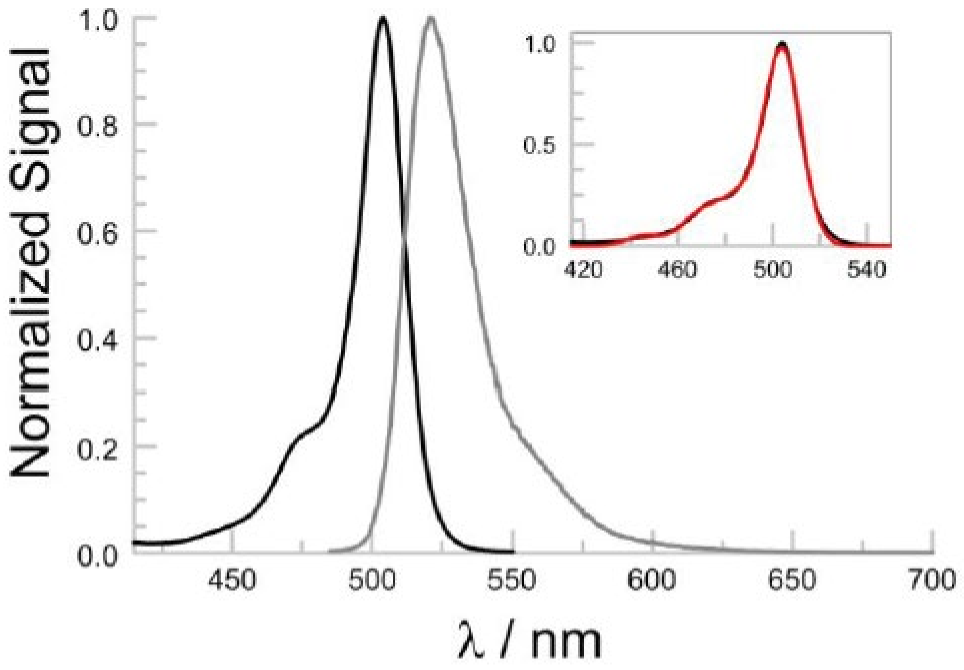

Figure S21. Normalized absorption (black curve) and fluorescence (grey curve) recorded for B-P_2-B in CH_2Cl_2 solution. The insert compares absorption (black curve) and excitation (red curve) spectra recorded in dilute solution.

Equations used to calculate spectroscopic parameters for the chromophores in dilute solution are as follows:

Huang-Rhys factor (S_M)

The intensities of the transitions between the vibrational ground state 0 in the electronic excited state and the vibrational level m in the electronic ground state are given by

$$I_{m.j} = \frac{e^{-S_J}(S_J)^m}{m!} \qquad (S1)$$

where the index j51,2,3,... denotes different vibrational modes belonging to the vibronic bands m51,2,3,... and Sj is the Huang–Rhys factor for vibrational mode j.

Re-organization energy (L)

$$L = \frac{SS}{2} = S_M h\nu \qquad (S2)$$

Where SS refers to the Stokes shift in units of cm^{-1} and hν is the medium-frequency vibronic mode coupled to Franck-Condon absorption or emission in units of cm^{-1}. The latter is obtained from Gaussian deconstruction of the reduced spectrum.

Reduced absorption spectrum

$$A(\nu) = \frac{\varepsilon(\nu)}{\nu} \qquad (S3)$$

Where ε is the molar absorption coefficient at a particular wavenumber ν.

Reduced fluorescence spectrum

$$F(\nu) = \frac{I(\nu)}{\nu^3} \qquad (S4)$$

Where I(ν) refers to the normalized emission intensity at wavenumber ν.

Transition dipole moment from absorption spectrum

$$\mu_{TD}^2 = \frac{3\varepsilon_0 hc ln(10)}{20\pi^2 N_A} \cdot \frac{9n}{(n^2+2)^2} \cdot \int \frac{\varepsilon}{\nu} d\nu \qquad (S5)$$

Where n is the refractive index of the surrounding medium, ε_0 is the vacuum permittivity and ε is the molar absorption coefficient at wavenumber ν, in units of M^{-1} cm^{-1}.

Transition dipole moment from fluorescence spectrum

$$\mu_{TD}^2 = \frac{3\varepsilon_0 h k_{RAD}}{16 \times 10^6 \pi^3} \cdot \frac{9}{n(n^2 = 2)^2} \cdot \frac{\int \frac{F(\nu)}{\nu^3} d\nu}{\int F(\nu)\, d\nu} \qquad (S6)$$

Where F(ν) refers to the normalized fluorescence intensity at wavenumber ν.

Spectral overlap integral

$$\int A(\nu) = A = 1 : \int F(\nu) = B = 1 \qquad (S7a)$$

$$\psi = \int A \times B\, d\nu \qquad (S7b)$$

Here A refers to the reduced absorption profile for the lowest-energy transition with the total area being normalized to unity. B refers to the corresponding emission spectral profile.

Rate constant for electronic energy transfer between the terminals

$$k_{DD} = \left[\frac{s\kappa}{4\pi\varepsilon_0} \cdot \frac{(\mu_{TD})^2}{R_{CC}^3}\right]^2 \cdot \frac{\psi}{\hbar^2 c} \qquad (S8a)$$

$$s = \frac{3}{2n^2+1} \qquad (S8b)$$

$$\kappa = cos\theta - 3cos\phi_A\phi_B \qquad (8c)$$

Here, n refers to the refractive index of the solvent. For the orientation factor, θ refers to the angle between the two transition dipole moment vectors while ϕ refers to the angle between the transition dipole moment vector and the molecular axis connecting the centers of the two BODIPY residues. The latter is illustrated on Chart S1.

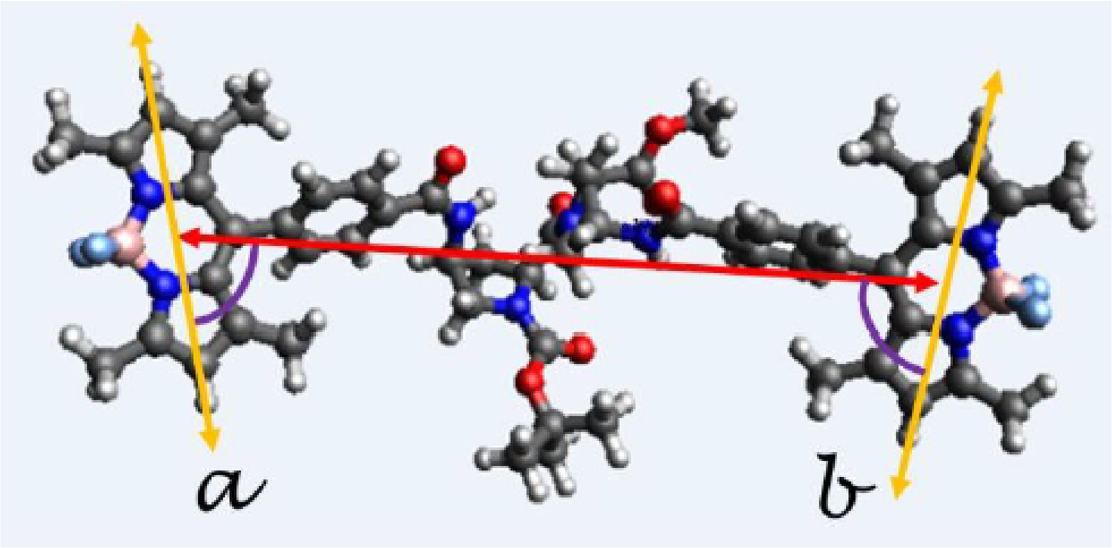

Chart S1. Pictorial representation of the orientation factor used to compute the rate constant for intramolecular dipole-dipole electronic energy transfer between the terminals of the symmetrical dimer. The transition dipole moment vectors for the two BODIPY residues are illustrated as yellow lines while the imaginary line connecting the two centers is shown in red. The required angles are shown as indigo arcs.

Kasha model for excitonic coupling

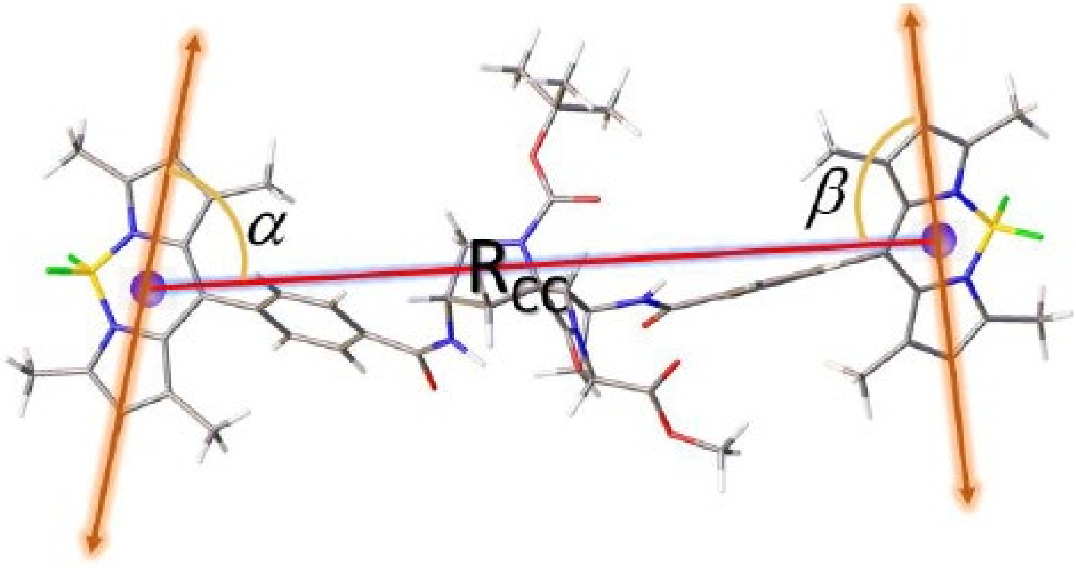

Figure S22. Illustration of the structural parameters associated with the Kasha model for intermolecular excitonic coupling.

$$J_D = \frac{1}{4\pi\varepsilon_0} \cdot \frac{\mu_{TD}^2}{100hcR_{CC}^3}(1 - 3cos^2\theta) \qquad (S9a)$$

$$\theta = 180 - \alpha - \beta \qquad (S9b)$$

Here, J_D refers to the magnitude of the excitonic coupling interaction energy according to the Kasha model. The transition dipole moment vectors associated with the BODIPY chromophores are illustrated as brown lines running along the long molecular axis. A dummy atom (blue sphere) is used to indicate the center of each chromophore. The respective center-to-center distance (R_{CC}) is indicated as a red line. The angle (θ) between the two transition dipole moment vectors cannot be obtained directly but becomes accessible using the angles between the vectors and the intermolecular axis. For the symmetrical dimer, α = 76.9° and β = 82.8°. For the pseudo-dimer, α = 77.5° and β = 87.5°.

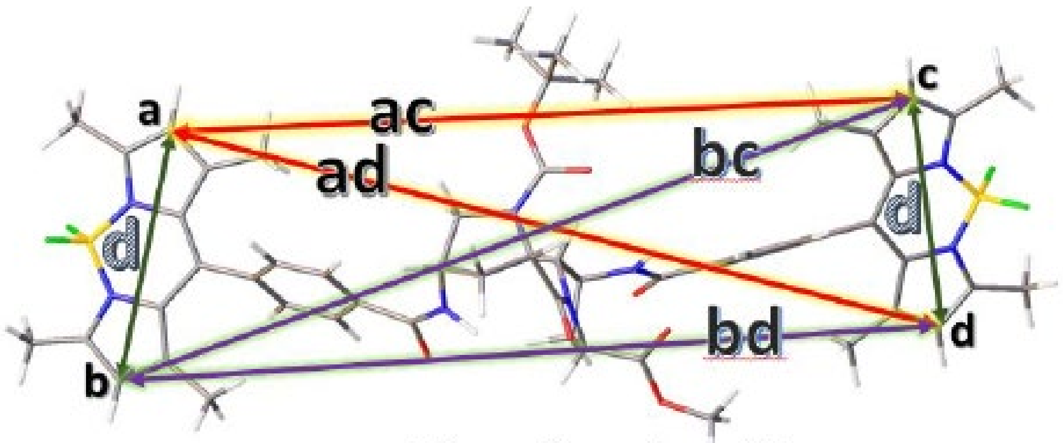

$$f(d) = \left[\frac{1}{ac} + \frac{1}{bd} - \frac{1}{ad} - \frac{1}{bc}\right]$$

Figure S23. Illustration of the geometry factors associated with the Kuhn model for intermolecular excitonic coupling.

$$J_K = \frac{\mu_{TD}^2}{4\pi\varepsilon_0 d^2} \cdot f(d) \qquad (S10)$$

Here J_K refers to the magnitude of excitonic coupling interaction energy between the two chromophores according to the Kuhn model. The distance parameter is illustrated by way of Figure S21 while d (= 6.75 Å) represents the dipole length. The effect of solvent screening is taken into account during formulation of the transition dipole moment.

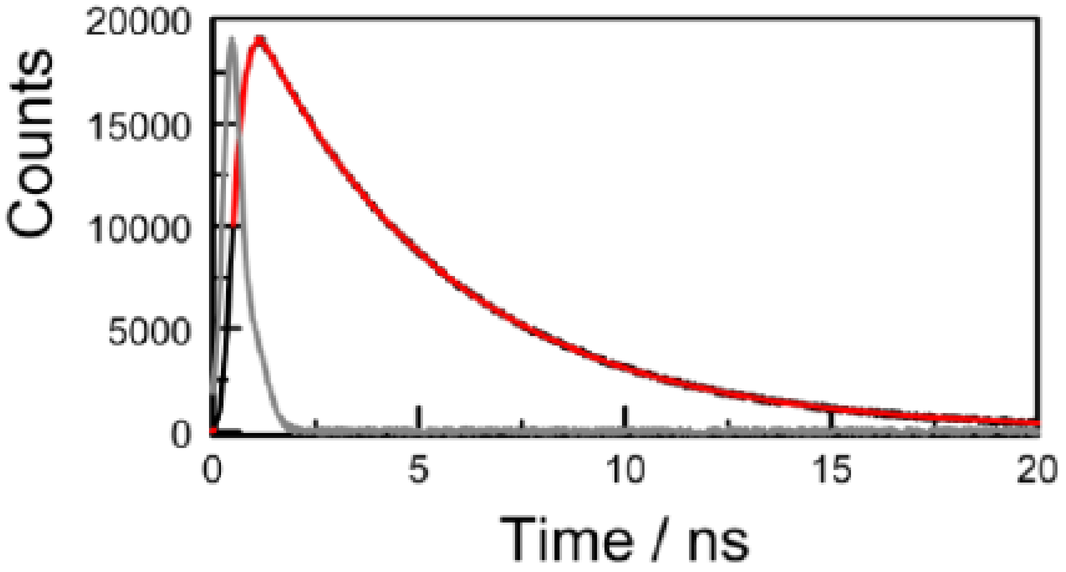

Figure S24. Time-resolved fluorescence decay curve recorded for P-B_1 in CH_2Cl_2 solution following excitation at 440 nm. The experimental decay trace is shown as a black curve while the statistical fit to a lifetime of 4.8 ns is shown in red. The instrumental response function is shown as a grey curve.

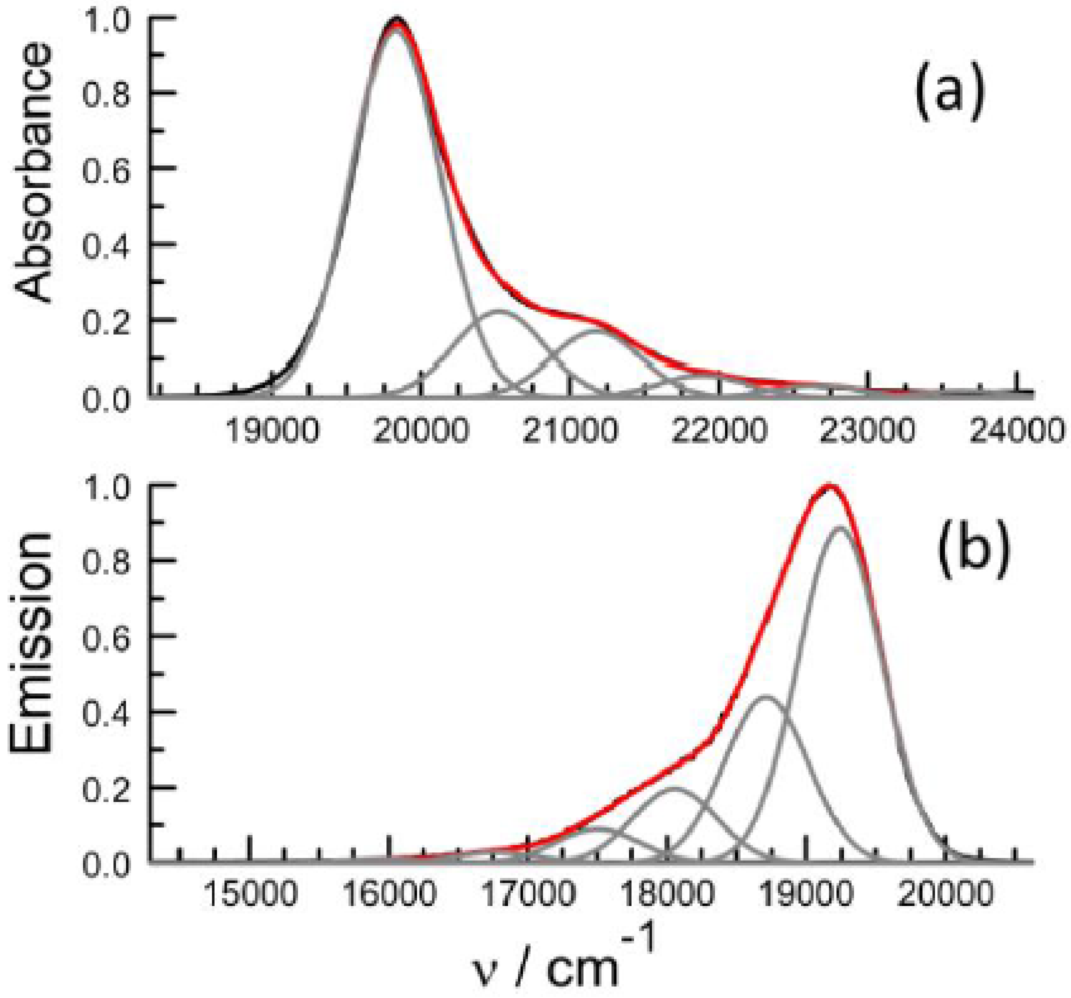

Figure S25. (a) Reduced absorption and (b) reduced fluorescence spectra recorded for B-P_2-B in CH_2Cl_2 solution. In each case, the experimental spectrum is shown as a black curve with the simulated spectrum superimposed as a red curve. The individual Gaussian components used to compile the vibronic manifold are shown as grey curves.

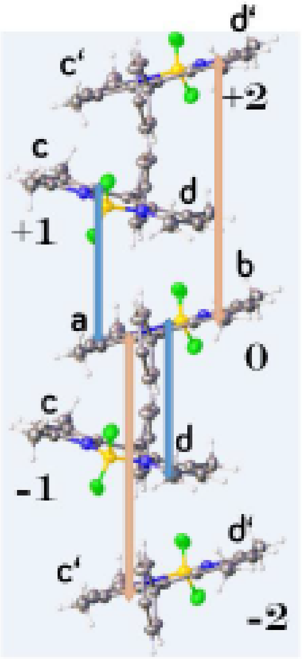

$$J_D = 1045 \times \mathcal{F}(d)$$

$$\mathcal{F}(d) = \left[\left(\frac{1}{ac}\right) + \left(\frac{1}{bd}\right) - \left(\frac{1}{ad}\right) - \left(\frac{1}{bc}\right)\right]$$

Figure S26. Illustration of the mutual excitonic coupling between BODIPY chromophores located along the same filament. The reference chromophore is labelled as "0" while chromophores sited immediately above (+1) and below (-1) are easily identified. The next layers of chromophores are designated as ± 2, etc. The magnitude of excitonic coupling is then computed for each pair of chromophores from the given above. Here, J_D is given in units of cm^{-1}. For the distance factor, individual distances are measured for carbon atoms located at the ends of the transition dipole moment vectors, with the reference chromophore being designated as a and b. The second chromophore is designated as c and d. All distances are taken from the crystal structure.

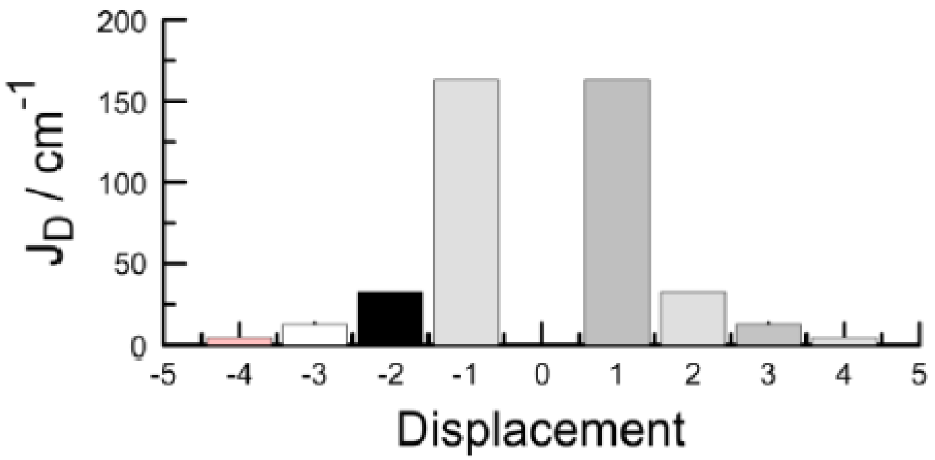

Figure S27. Illustration of how the excitonic coupling strength depends on the location of the second chromophore along the filament. See Figure S26 for an explanation of the positions of the chromophores.

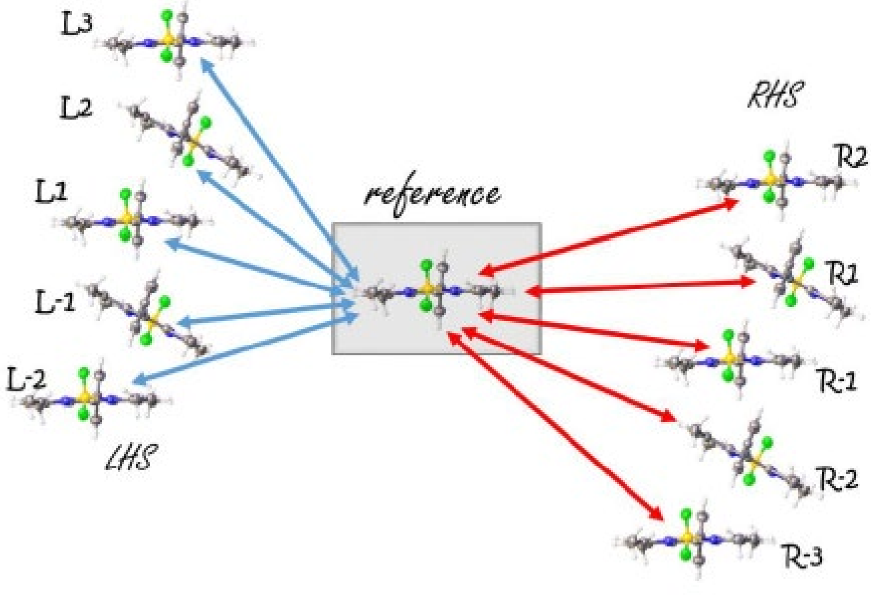

Figure S28. Illustration of excitonic coupling between a reference chromophore and BODIPY molecules located in adjacent columns. To allow for geometrical asymmetry, the adjacent columns are labelled as left-hand side (LHS) and right-hand side (RHS). Individual chromophores are assigned a positional number according to their location relative to the reference. The excitonic coupling is then calculated for each pair of chromophores.

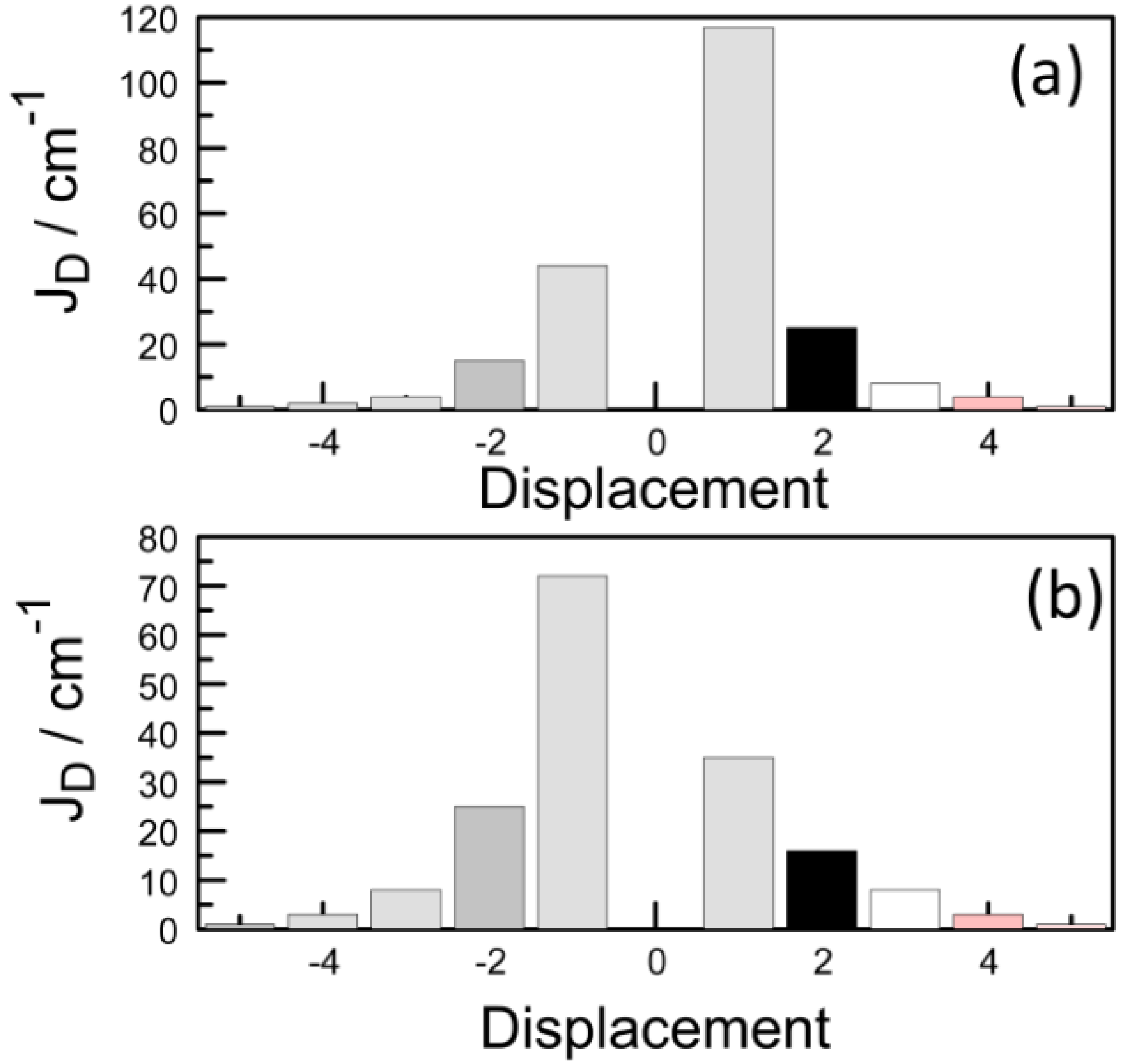

Figure S29. Illustration of how the excitonic coupling strength depends on the location of the second chromophore located on an adjacent column. Panel (a) refers to the RHS and panel (b) refers to the LHS. See Figure S28 for an explanation of the position of each chromophore.

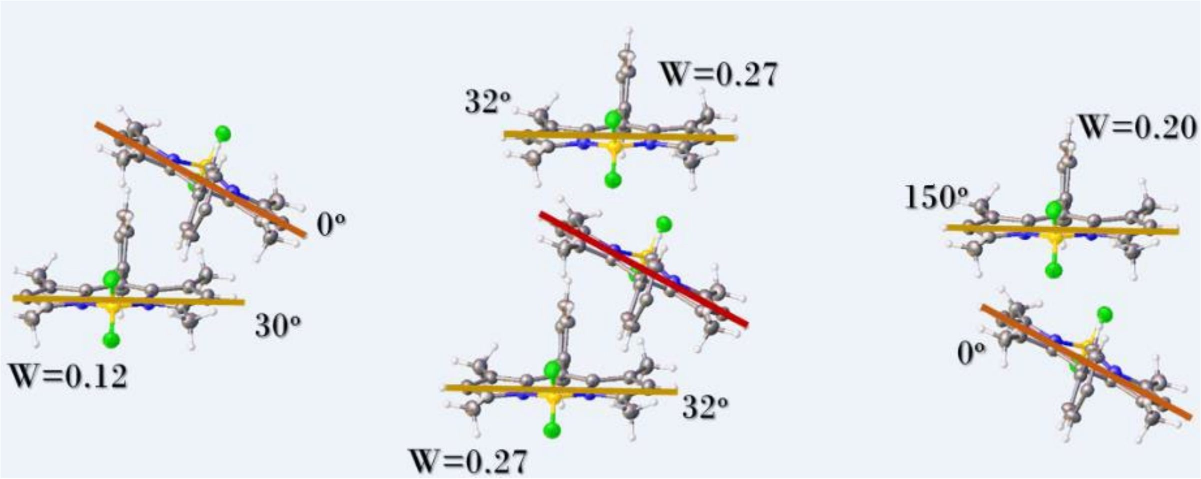

Figure S30. Illustration of the calculation used to determine the mean angle (α) between the transition dipole moment vectors on the reference chromophore (central BODIPY, TDMV marked in red) and the closest six chromophores. The experimentally determined angle is shown for each chromophore. The weighting factor, W, is shown for each of the surrounding six BODIPYs. This was calculated by dividing the J_D derived for any particular chromophore by the total J_D for the six pairs of chromophores. The final angle was found as the sum of individual products of αW. Note, two of the six angles are close to zero and do not participate in the calculation, except in terms of establishing the W values.

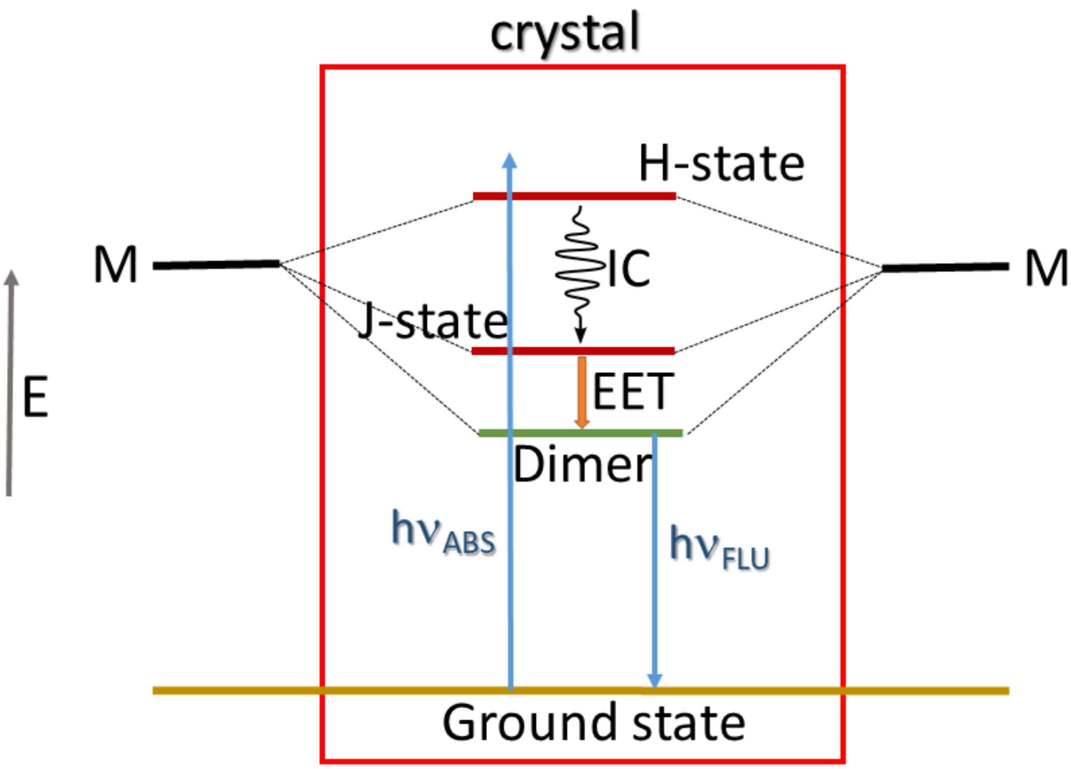

Figure S31. Energy level diagram proposed for B-P_1 on passing from the monomer (M) in solution to the crystalline phase. Excitonic splitting for an oblique set of chromophores causes formation of H- and H-states. Dimerization at the interface gives rise to a strongly fluorescent aggregate responsible for the red emission. The latter state is populated by fast electronic energy transfer (EET) from the J-state while rapid internal conversion (IC) results in quantitative conversion of the H-state to the lower-energy J-state.

S4. Reflectance spectrum recorded for crystalline B-P_1

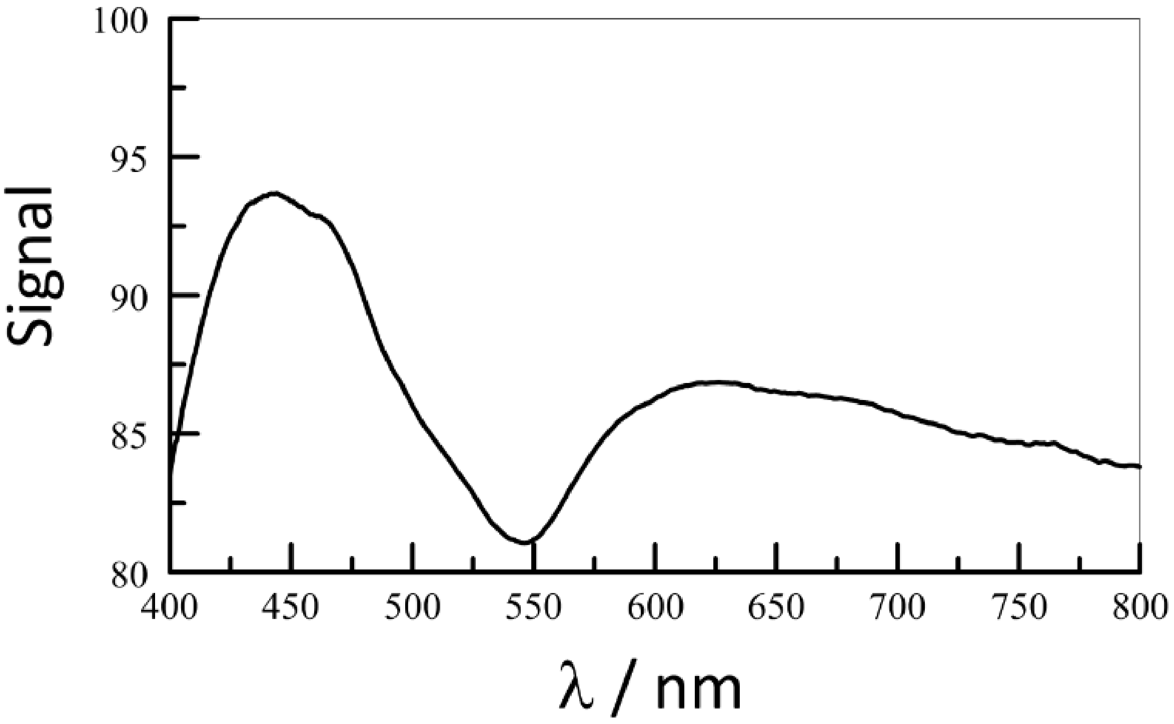

Figure S32. Reflectance spectrum recorded for a single crystal of B-P_1. Steady-state absorption spectra for single crystals of B-P_1 were measured in reflectance mode. To this end an Ocean Optics USB 2000+ spectrophotometer was used with a proprietary fibre optic attachment to integrate light scattered from the sample immobilized on a quartz coverslip. The instrument was calibrated by means of a reflectance standard, Spectralon polytetrafluoroethylene (PTFE).
